# Classification of Peripheral Blood Leukocyte Phenotypes and Serum Cytokines in Vogt–Koyanagi–Harada Disease before and after Glucocorticoid Therapy

**DOI:** 10.3390/jcm12247742

**Published:** 2023-12-17

**Authors:** Tomohito Sato, Nanae Taniguchi, Yoshiaki Nishio, Masataka Ito, Masaru Takeuchi

**Affiliations:** 1 Department of Ophthalmology, National Defense Medical College, Tokorozawa, Saitama 359-8513, Japan; dr21043@ndmc.ac.jp (T.S.); esahc621hjjnan@gmail.com (N.T.); cln349@ndmc.ac.jp (Y.N.); 2 Department of Developmental Anatomy and Regenerative Biology, National Defense Medical College, Tokorozawa, Saitama 359-8513, Japan; masataka@ndmc.ac.jp

**Keywords:** cytokine, glucocorticoid, leukocytes, mass cytometry, multiplex bead analysis, Vogt–Koyanagi–Harada disease

## Abstract

Vogt–Koyanagi–Harada disease (VKH) is an autoimmune disease, and glucocorticoid therapy (GC) is widely used for VKH. We provided a profile of leukocyte populations and serum cytokines in VKH patients under GC. A prospective observational study was conducted on three treatment-naïve VKH patients. Peripheral blood samples were collected from the patients before GC (VKH-acute) and after 6 months (VKH-remission), and healthy individuals were used as controls. Proportions of 37-type leukocytes and levels of 27-kind cytokines were measured by mass cytometry and multiplex bead analysis. Property similarity was analyzed using hierarchical cluster analysis. The leukocytes and cytokines were broadly classified into four and three clusters: (1) a cluster with high intensity in VKH-acute consisting of B cells, Th2-like, Th17-like, basophils, and IL-7 and IP-10; (2) a cluster with high intensity in VKH-remission composed of monocytes, neutrophils, IL-4, and TNFα; in leukocytes, (3) a cluster with low intensity in VKH-acute and -remission consisting of CD8^+^ T cells, Th1-like, and NKT cells; (4) a cluster with low intensity in VKH-remission composed of NK cells, Tregs, and DCs; and in cytokines, (5) a cluster with high intensities in VKH-acute and -remission comprising G-CSF, MCP-1, eotaxin, and IL-17A. These findings suggest that inflammatory composition in blood during the acute phase of VKH represents complex hyperimmune responses dominantly driven by Th and B cells.

## 1. Introduction

Vogt–Koyanagi–Harada (VKH) disease is one of the most common uveitis [1]. Uveitis is the leading cause of vision loss and accounts for an estimated 25% of all blindness cases in Western and developing countries [2]. VKH is a multisystem T lymphocyte-mediated autoimmune disease against melanocytes expressing HLA-DR, which is present as an antigen in the target organs including the uvea, inner ear, meninges, and integumentary system [3]. Memory T cell subsets such as cytotoxic T cells [4], T helper type 1 (Th1) cells [5,6], and T helper type 17 (Th17) cells [6,7] have been identified as pathogenic immune cells of VKH disease. In addition to the memory T cell subsets, other immune cells, including natural killer (NK) cells, B cells, and myeloid cells, are also involved in the pathology of VKH disease [8].

Tissue-resident dendritic cells (DCs) and macrophages are the major professional antigen-presenting cells, and monocytes are regarded as their counterparts in peripheral blood [9,10]. Monocytes release proinflammatory cytokines, such as interleukin (IL)-6, IL-1β, and TNFα, thereby directly mediating inflammation [10]. At present, human monocytes are generally classified into three subpopulations based on the differential expression levels of CD14 and CD16 [11], and each subpopulation exhibits the following characteristic functions: (1) classical monocytes, which perform better in phagocytosis compared to other monocyte subsets, and contribute to antimicrobial responses [12,13,14], (2) intermediate (transitional) monocytes, which are specialized in antigen processing and presentation [13,15], and (3) non-classical monocytes, which have the capacity to patrol blood vessels, resist viruses, and stimulate T cell proliferation [16,17]. Although previous studies indicate the participation of certain immune cells in the destruction of melanocyte-enriched organs of VKH disease patients, the underlying mechanism of the disease remains to be defined [9].

Glucocorticoid (GC) therapy is widely used as the first-line treatment for VKH disease in the acute phase because almost all patients respond well and achieve amelioration of symptoms within a few days [9]. GC therapy directly or indirectly inhibits T cell activation, promotes apoptosis in immune cells, and exerts inhibitory effects on inflammatory mediators in VKH patients [9]. However, the pathogenic roles of leukocytes and their comprehensive involvement in systemic immunity before and after GC therapy in VKH disease patients have not been well addressed.

This study was designed to elucidate a comprehensive profile of leukocytes in peripheral blood and serum cytokines of VKH disease patients using mass cytometry and multiplex bead analysis. In the present study, we evaluated the property similarities among the leukocytes and the cytokines in the acute and remission phases of VKH disease patients to explore their relationships with the pathology of VKH disease.

## 2. Materials and Methods

### 2.1. Subjects

This prospective observational study was performed on 3 treatment-naïve VKH disease patients who received high-dose intravenous GC pulse therapy followed by tapering with oral GC therapy at the National Defense Medical College Hospital between 1 December 2020 and 31 July 2022. Seven healthy volunteers not matched for age were recruited from the employees of the hospital as relative controls.

Peripheral blood samples were collected from the patients before the GC pulse therapy (acute phase) and approximately 6 months later under low-dose oral GC therapy (remission phase) at a daily dose of 2.5 mg prednisolone in one patient and 5.0 mg in the other two patients. The controls underwent peripheral blood sampling once.

The study protocol was reviewed and approved by the Ethics Committee of the National Defense Medical College (Clinical Ethics Approval No. 4210 on 28 July 2020), and the procedures conformed to the tenets of the Declaration of Helsinki. Written informed consent was obtained from all VKH disease patients and the volunteers before entry.

### 2.2. Diagnostics and Treatments 

Diagnosis of VKH disease and classification of the disease types were conducted according to a report by the International Committee on Nomenclature [18]. Three uveitis specialists (members of the Japanese Ocular Inflammation Society) reviewed the clinical findings of the VKH disease patients and confirmed the diagnosis and disease classification. Results of the best-corrected visual acuity test using a decimal chart were converted to a logarithm of the minimum angle of resolution (logMAR) units for statistical analysis. Central foveal thickness (CFT) was defined as the minimum distance between the internal limiting membrane and the anterior boundary of the retinal pigment epithelium layer [19]. Central retinal thickness (CRT) was defined as the mean retinal thicknesses of a central 1 mm circle on the Early Treatment Diabetic Retinopathy Study grid in the macula [20]. Subfoveal choroidal thickness (SFCT) was determined as the whole-layer thickness of the choroid below the fovea [21]. Data from the right eye were used for comparisons of ophthalmologic test values.

### 2.3. Leukocyte Phenotypes and Their Proportions in Peripheral Blood

Peripheral blood samples were collected in heparinized vacutainer tubes. The samples were transported to St. Luke’s SRL Advanced Clinical Research Center for measurement, data processing, and analysis of leukocytes. The leukocyte phenotypes and the proportions of immune cells in peripheral blood were measured by time-of-flight technology using mass cytometry (Helios^TM^; Fluidigm, South San Francisco, CA, USA) and the Maxpar Direct Immune Profiling Assay^®^ (Fluidigm, South San Francisco, CA, USA) [22]. The leukocytes were classified into 37 types of immune cells based on differentiation and function as well as maturation stage, as follows: lymphocytes, cluster of differentiation (CD) 3^+^ T cells, CD8^+^ T cells, naïve CD8^+^ T cells, central memory CD8^+^ T cells, effector memory CD8^+^ T cells, terminal effector CD8^+^ T cells, CD4^+^ T cells, naïve CD4^+^ T cells, central memory CD4^+^ T cells, effector memory CD4^+^ T cells, terminal effector CD4^+^ T cells, regulatory T cells (Tregs), Th1-like, T helper type 2 (Th2)-like, Th17-like, gamma delta (γδ) T cells, mucosal-associated invariant T (MAIT)/natural killer T (NKT) cells, B cells, naïve B cells, memory B cells, plasmablasts, natural killer (NK) cells, NK cells early, NK cells late, monocytes, classical monocytes, transitional monocytes, non-classical monocytes, DCs, plasmacytoid DCs (pDCs), myeloid DCs (mDCs), granulocytes, neutrophils, basophils, eosinophils, and CD66b^−^ neutrophils.

Whole blood staining, sample acquisition, and data normalization were performed according to the manufacturer’s instructions [22]. The flow cytometry standard files generated by Helios^TM^ were analyzed by the Maxpar Pathsetter, an automated analysis system powered by GemStone^TM^ 3.0.15 (Verity Software House, Topsham, ME, USA) [22]. 

### 2.4. Serum Cytokines Levels

Peripheral blood samples for detecting levels of serum cytokines were collected at the same time as the mass cytometry specimens. The blood samples were centrifuged at 3000 rpm for 10 min, and the serum component was stored at −80 °C until measurement. The levels of serum cytokines were measured using a human-premixed multi-analyte kit (Bio-Plex Human Cytokine 27-plex panel; Bio-Rad, Hercules, CA, USA). Twenty-seven types of cytokines were assayed as follows: platelet-derived growth factor-BB, interleukin (IL)-1β, IL-1 receptor antagonist (ra), IL-2, IL-4, IL-5, IL-6, IL-7, IL-8, IL-9, IL-10, IL-12, IL-13, IL-15, IL-17A, eotaxin, basic fibroblast growth factor, granulocyte colony-stimulating factor (G-CSF), granulocyte macrophage colony-stimulating factor, interferon-gamma (IFN-γ), IFN-γ-inducible protein 10 (IP-10), monocyte chemotactic protein-1 (MCP-1), macrophage inflammatory protein (MIP)-1α, MIP-1β, regulated on activation, normal T cell expressed and secreted (RANTES), tumor necrosis factor-alpha (TNFα), and vascular endothelial growth factor (VEGF)-A.

All standards and samples were measured in duplicate. Serum cytokine levels below detectable levels were treated as 0 in statistical analysis [23].

### 2.5. Statistical Analysis

Statistical analyses were performed using the statistic add-in software for Excel (BellCurve for Excel^®^, SSRI Co., Ltd., Tokyo, Japan; and XLSTAT^®^, Addinsoft Company, Paris, France). Fisher’s exact test (for *n* < 4) was used to compare categorical variables. A Wilcoxon *t*-test and a Mann–Whitney *U* test were used for nonparametric comparisons between two paired groups and between two unpaired groups, respectively. Hierarchical cluster analysis was performed using Ward’s method with Euclidean distance as the distance metric [23]. The mean value was used as the assigned value for the hierarchical cluster analysis. A two-tailed *p*-value of less than 0.05 was considered to be statistically significant. 

In subsequent hierarchical cluster analysis, we performed a priori power calculation using clinical data of the present study. We calculated effect sizes (Hedges’ g) for the proportion of CD8^+^ T cells in peripheral blood (VKH disease patients: *n* = 3, percentage in the acute phase; 5.35 (mean) ± 2.04 (standard deviation), percentage in the remission phase; 3.04 ± 1.44, healthy individuals as relative controls: *n* = 7, 13.3 ± 4.09), which is a representative immune cell with significant difference between VKH disease patients and healthy controls [9]. The effect sizes for CD8^+^ T cells in the acute and remission phases of VKH disease patients were 2.16 and 2.84, respectively. To confirm significant differences in the proportion of CD8^+^ T cells with a statistical power of 0.80 [24], the sample size of our study would be 4.3 for the two-tailed comparison between VKH disease patients in the acute phase and controls and 2.9 for the comparison between VKH disease patients in the remission phase and controls. Therefore, we attempted to recruit 3 or more cases in order to allow some margin in the population of VKH disease and control groups.

## 3. Results

### 3.1. Subjects

The demographics of VKH disease patients and relative controls, as well as the clinical data of the patients, are shown in Table 1. All three patients were diagnosed with incomplete VKH disease based on ocular features and laboratory findings [18]. The median age was 66 years (range 54–75 years) in the VKH disease group and 30 years (range 23–45) in the control group. The VKH disease group was older than the control group (*p* = 0.038). The male-to-female ratio was 1:2 in the VKH disease group and 4:3 in the control group, with no significant difference between the two groups. In comparison between the acute phase and remission phases of the VKH disease groups, there were no significant differences in logMAR VA, intraocular pressure, CFT, CRT, and SFCD between the two groups.

The clinical course of each VKH disease patient under GC therapy is shown in Supplementary Figures S1–S3.

### 3.2. Profile of Leukocyte Phenotypes and Proportions in Peripheral Blood 

**Figure 1:**
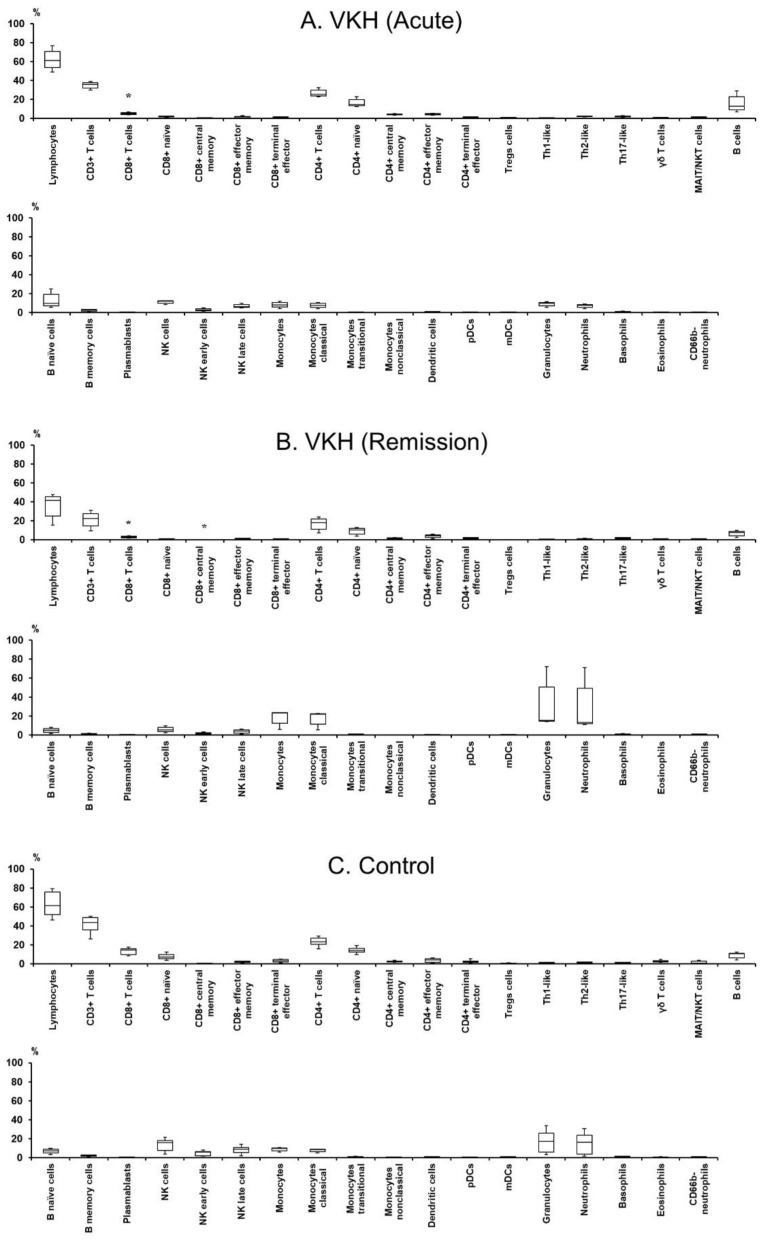
Histograms of immune cell populations, phenotypes, and proportions among leukocytes in the peripheral blood of VKH disease patients and controls. The histograms are composed of 37 types of immune cell populations among leukocytes in the VKH disease patient group before ((**A**); VKH acute) and after glucocorticoid therapy ((**B)**; VKH remission) and the control group (**C**). The vertical axis indicates the proportions of immune cells, and the horizontal axis shows immune cell populations and phenotypes. The bottom and top ends of the boxplots indicate the 25th and 75th percentiles. The bottom and top whiskers indicate the 10th and 90th percentiles. A solid line within each boxplot marks the median. VKH disease patients in the acute phase are the same as VKH patients in the remission phase. The number of patients is three. The number of controls is seven. Cell proportions are expressed in percentiles. Comparisons between VKH disease and control groups were evaluated by a Mann–Whitney *U* test. Comparisons between acute and remission phases in the VKH disease groups were performed by a Mann–Whitney *U* test. The *p*-value is two-tailed. VKH; Vogt–Koyanagi–Harada. *: *p* < 0.05.

Immune cell populations, phenotypes, and proportions among leukocytes in the peripheral blood of VKH disease patients and relative controls were determined to examine the complex involvements of leukocytes in the pathology of VKH disease (Table 2 and Table 3, Figure 1). The proportion of CD 8^+^ T cells in the acute and remission phases of VKH disease patients was lower than relative controls. The proportion of central memory CD8^+^ T cells in the remission phase of VKH disease patients was low compared to relative controls. There were no significant differences in the proportions of all the leukocyte populations between the acute and remission phases of VKH disease patients.

Profiles of leukocyte phenotypes and proportions in the peripheral blood of each VKH disease patient and healthy control are presented in Supplementary Table S1 and Supplementary Table S2, respectively. Hematologic data in each VKH disease patient are shown in Supplementary Tables S3–S5.

### 3.3. Hierarchical Cluster Analysis of Immune Cell Populations among Leukocytes in Peripheral Blood

**Figure 2:**
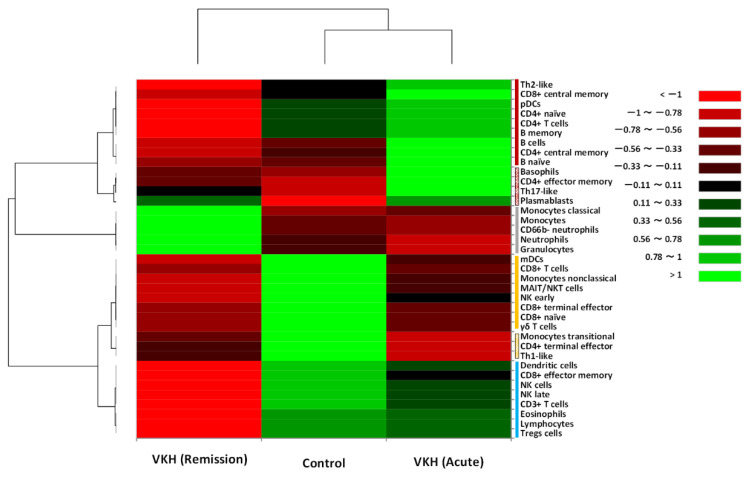
Classification of immune cell populations and phenotypes among leukocytes in peripheral blood by hierarchical cluster analysis. The heat map is composed of 37 types of immune cell populations among leukocytes in the VKH disease patient group before (VKH acute) and after glucocorticoid therapy (VKH remission) and the control group. The cell populations and phenotypes are broadly classified into four clusters based on property similarity: (1) Cluster A (red bar), (2) Cluster B (gray bar), (3) Cluster C (yellow bar), and (4) Cluster D (blue bar). Color scale: low values, red; middle-to-high values, black to green. The vertical axis indicates immune cell populations and phenotypes, and the horizontal axis shows the three groups.

Hierarchical cluster analysis was performed to classify immune cell populations among leukocytes in peripheral blood into groups with similar properties called clusters [25]. The leukocytes in the acute and remission phases of VKH disease patients and relative controls were broadly classified into four principal clusters (Figure 2, vertical bars on the right) as follows: (1) Cluster A (red bar), (2) Cluster B (gray bar), (3) Cluster C (yellow bar), and (4) Cluster D (blue bar). 

Cluster A consisted of a subcluster (filled red bar) formed by Th2-like, central memory CD8^+^ T cells, pDCs, naïve CD4^+^ T cells, CD4^+^ T cells, memory B cells, B cells, central memory CD4^+^ T cells, and naïve B cells; and another subcluster (patterned red bar) is composed of basophils, effector memory CD4^+^ T cells, Th17-like, and plasmablasts. The intensity of Cluster A in the acute phase of the VKH disease (VKH-acute) group was high compared to the remission phase of the VKH disease (VKH-remission) group and the control group. Cluster B was composed of classical monocytes, monocytes, CD66b^−^ neutrophils, neutrophils, and granulocytes. The intensity of Cluster B in the VKH-remission group was high compared to the VKH-acute and control groups. Cluster C consisted of a subcluster (filled yellow bar) formed by mDCs, CD8^+^ T cells, non-classical monocytes, MAIT/NKT cells, NK cells early, terminal effector CD8^+^ T cells, naïve CD8^+^ T cells, and γδ T cells; and another subcluster (patterned yellow bar) is composed of transitional monocytes, terminal effector CD4^+^ T cells, and Th1-like. The intensity of Cluster C in the control group was high compared to the VKH-acute and -remission groups. Cluster D was composed of DCs, effector memory CD8^+^ T cells, NK cells, NK cells late, CD3^+^ T cells, eosinophils, lymphocytes, and Tregs. The intensity of Cluster D in the VKH-remission group was low compared to the VKH-acute and control groups.

In summary, Th2 cell-, Th17 cell-, and B cell-dominant immunity was formed in the acute phase of VKH disease, and the immunity was shifted by GC therapy to classical monocyte- and neutrophil-dominant in the remission phase. 

### 3.4. Profile of Serum Cytokine Levels

**Figure 3:**
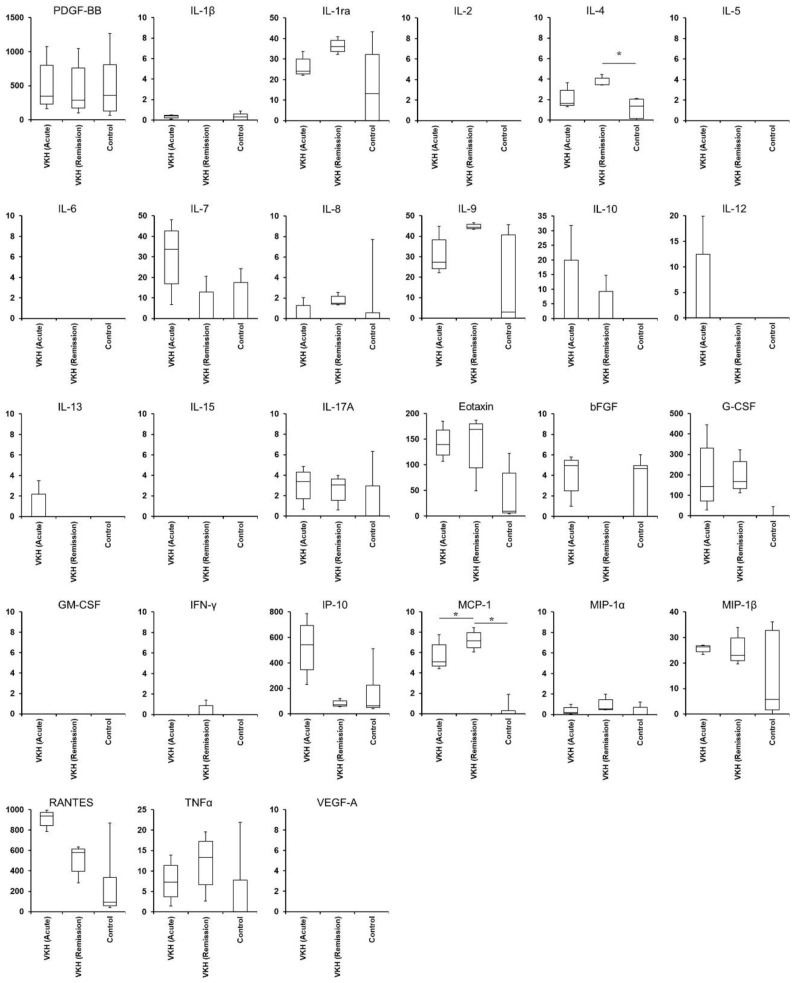
Histograms of serum cytokine levels in VKH disease patients and controls. Histograms show serum levels of 27 kinds of cytokines in the VKH disease patient group before (VKH acute) and after glucocorticoid therapy (VKH remission) and the control group. The vertical axis indicates levels of cytokines, and the horizontal axis shows the three groups. Cytokine levels are presented in units of pg/mL. Comparisons between VKH disease and control groups were evaluated by a Mann–Whitney *U* test. Comparisons between acute and remission phases in the VKH disease groups were performed by a Mann–Whitney *U* test. The *p*-value is two-tailed. *: *p* < 0.05.

Levels of serum cytokines affecting the proliferation and differentiation of leukocytes were described in Table 4 and Table 5 and Figure 3. The MCP-1 level in the acute and remission phases of VKH patients was high compared to relative controls. The IL-4 level in the remission phase of VKH patients was high compared to relative controls. There were no significant differences in all the measured cytokine levels between the acute and remission phases of VKH patients.

The serum cytokine levels in each VKH disease patient are shown in Supplementary Table S6. The serum cytokine levels in each healthy control are presented in Supplementary Table S7.

### 3.5. Hierarchical Cluster Analysis of Serum Cytokines

**Figure 4:**
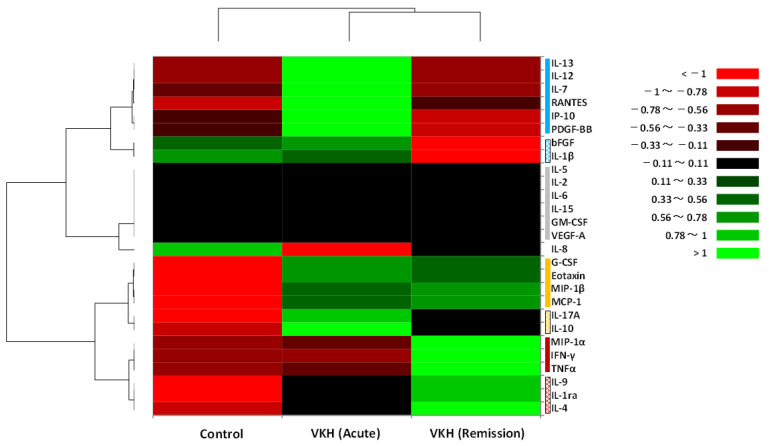
Classification of serum cytokines by hierarchical cluster analysis. The heat map is composed of 27 kinds of serum cytokines in the VKH disease patient group before (VKH acute) and after glucocorticoid therapy (VKH remission) and the control group. The cytokines are roughly divided into four clusters and a single cytokine based on property similarity: (1) Cluster A (blue bar), (2) Cluster B (gray bar), (3) Cluster C (yellow bar), (4) Cluster D (red bar), and (5) IL-8. The vertical axis indicates cytokines, and the horizontal axis shows the three groups.

Based on property similarity, serum cytokines in the VKH-acute, VKH-remission, and control groups were roughly divided into four primary clusters (Figure 4, vertical bars on the right) and a single cytokine as follows: (1) Cluster A (blue bar), (2) Cluster B (gray bar), (3) Cluster C (yellow bar), (4) Cluster D (red bar), and (5) IL-8.

Cluster A consisted of a subcluster (Cluster A-1, filled blue bar) formed by IL-13, IL-12, IL-7, RANTES, IP-10, and PDGF-BB, which stimulate activations of Th1, Th2, and B cells; and another subcluster (patterned blue bar) composed of bFGF and IL-1β that affect remodeling in the inflammation site. The intensity of Cluster A in the VKH-acute group was high compared to the VKH-remission group. Furthermore, the intensity of Cluster A-1 in the VKH-acute group was high compared to the control group. Cluster B was composed of IL-5, IL-2, IL-6, IL-15, GM-CSF, and VEGF-A. There were no differences in the intensities among the three groups because those cytokines were secreted in small quantities in all three groups (Table 4, Figure 3). Cluster C consisted of a subcluster (Cluster C-1, filled yellow bar) formed by G-CSF, eotaxin, MIP-1β, and MCP-1, which may induce allergic responses and the activation of innate immune cells; and another subcluster (Cluster C-2, patterned yellow bar) composed of IL-17A and IL-10 that induce and suppress activations of immune cells, respectively. The intensity of Cluster C-1 was almost the same in the VKH-acute and VKH-remission groups but was high compared to the control group. Furthermore, the intensity of Cluster C-2 in the VKH-acute group was the highest among the three groups, and the intensity in the VKH-remission group was high compared to the control group. Cluster D was composed of a subcluster (filled red bar) formed by MIP-1α, IFN-γ, and TNFα, which stimulated the activation of Th1-related immune responses; and another subcluster (patterned red bar) consisted of IL-9, IL-1ra, and IL-4 and could activate B cell-related immune and allergic responses. The intensity of Cluster D in the VKH-remission group was high compared to the VKH-acute and control groups.

In summary, Th cell- and B cell-related cytokines were relatively elevated both in the acute and remission phases of VKH disease, although cytokines depending on secretory cells were altered following the initiation of GC therapy. Furthermore, the activation of innate immune cells and allergic responses may be induced continuously during the acute and remission phases of VKH disease.

## 4. Discussion

The major findings of our study are summarized as follows, (1) A complex hyperimmune response induced predominantly by Th cells and B lymphocytes resembling Type IV hypersensitivity reactions may be the inflammatory composition in peripheral blood during the acute phase of VKH disease. (2) GC therapy may alter the systemic immune system of VKH disease from the activation of adaptive immunity to the activation of innate immunity. (3) Th17 lymphocytes and Th17-associated cytokines are relatively elevated both in the acute and remission phases of VKH disease and could be significant pathological factors in the disease.

VKH disease is an autoimmune disease, in which tyrosinase and tyrosinase-related protein-1 and -2 of melanocytes are primarily identified as autoantigens [26]. The dysregulation of the T cell compartment is a well-known hallmark of autoimmune diseases [9], and Th1/Th17 polarization may play a major role in the pathology of VKH disease [6,7,27]. Alternatively, the potential role of B lymphocytes in the pathology has been exemplified in some studies [3,28]. Furthermore, Jiang et al. [9] reported that the proportion of NK cells decreased, and the proportion of monocytes increased in the acute phase of VKH disease patients, while there were no significant differences in the absolute numbers and proportions of T cells and B lymphocytes between VKH patients and controls. These seemingly contradictory findings in previous reports suggest that VKH disease is a multifactorial disease involving multiple immune cells simultaneously, and it would be inappropriate to explain the complex inflammatory composition in peripheral blood during the acute phase of VKH disease based on a distinct inflammatory mechanism against specific autoantigens.

Hypersensitivity reactions are closely related to the etiology of autoimmune diseases [29]. “Allergy”, which is almost synonymous with “hypersensitivity”, was first used by von Pirquet to denote both host-protective and potentially host-injurious immune responses [30,31]. In the Gell–Coombs system, hypersensitivity is roughly classified into four types, and they are briefly described as follows: (1) Type I; IgE antibody-mediated anaphylaxis against mast cells, (2) Type II; cytotoxic reactions mediated by IgG or IgM antibodies, (3) Type III; cytotoxic reactions mediated by antigen-antibody complexes with complement, and (4) Type IV; delayed cellular reactions mediated by CD4^+^ cells and CD8^+^ cells [29].

VKH disease is a bilateral, chronic, and granulomatous uveitis [3]. Inomata and Sakamoto [32] identified an immunohistochemically scattered infiltration of T cells and B lymphocytes in the thickened choroid in autopsy eyes of VKH disease patients. Recently, Abu El-Asrar et al. [3] postulated a potential mechanism of development in VKH disease as follows. (1) The autoantigen is a melanocyte-specific peptide [33], and molecular mimicry together with remnant epitopes from an unknown virus containing autoantigen [3,28] could elicit T cell and B lymphocyte responses through their cognate receptors, and (2) in the effector phase, overactive immune responses are driven by activated T cells and B lymphocytes [29]. Hammer [34] reported the involvement of cellular hypersensitivity to uveal pigment in VKH disease patients. In our study, the hierarchical cluster analysis of leukocyte phenotypes (Figure 2) demonstrated that the intensities of Th2-like, Th17-like, B cells, and basophils in Cluster A were high (brilliant green) in the acute phase of VKH disease patients compared to relative controls (dark red, black to dark green), whereas the intensity of Th1-like in Cluster C (dark red) was low compared to relative controls (brilliant green). These findings may support the hypothesis that the immune state in the acute phase of VKH disease is shifted to Th2-dominant immunity, which is responsible for the onset of hypersensitive responses [35]. Therefore, the inflammatory composition in peripheral blood during the acute phase of VKH disease may represent a component of the complex immune responses based on Type IV hypersensitivity.

With regard to methodology, a statistical method to examine property similarity among disease-related biomarkers, including leukocytes, cytokines, and mRNA, may be a useful tool not only to understand the characteristics of pathological immune responses but also to explore the immunotherapeutic effects of molecular-targeted therapies. These therapies include adalimumab [36] (an anti-TNFα receptor antibody approved for VKH disease) as well as other promising agents currently unapproved for VKH disease, such as rituximab [3] (an anti-CD20 monoclonal antibody that depletes B cells) and tofacitinib [37] (a Janus kinase-targeting inhibitor of T cell activation by downregulating cytokines secreted by Th17 cells [38]). In addition, Papasavvas et al. [39] reviewed the incidence proportions of chronic evolution and sunset-glow fundus in VKH disease patients following steroid monotherapy or steroid and non-steroid immunosuppressive therapies. In the VKH disease group following steroid monotherapy, chronic evolution and sunset fundus occurred in 44% and 59%, respectively, whereas the incidence of those were 2.3% and 17.5% in VKH disease patients under steroid and non-steroid immunosuppressive therapies [39]. Therefore, the statistical method could be an aid to elucidate the predictive biomarkers for prolongation of VKH disease.

Although numerous drugs have been developed in the last few decades, GC therapy remains the most widely used treatment for the acute phase of VKH disease because most patients respond well and achieve symptom amelioration within a few days [37,40]. Immune responses to GC are likely to be cell type-specific and may even be in the direction of transcriptional regulation [37,41]. By inhibiting T cell activation and promoting immune cell apoptosis, the primary benefit of GC for patients is the direct or indirect suppressive effects on T cells [42]. Jiang et al. [9] reported that GC therapy elevated the absolute number and proportion of granulocytes and decreased the proportion of lymphocytes, especially T cells and NK cells, in the peripheral blood of VKH disease patients. In this study, the hierarchical cluster analysis (Figure 2) indicated that GC therapy shifted the systemic immune state in VKH disease patients from lymphocyte-dominant immunity (Cluster A in the acute phase) to granulocyte (especially classical monocytes and neutrophils)-dominant immunity (Cluster B in the remission phase). On the other hand, the intensities of serum cytokine levels (Figure 4) indicated that Th1-related cytokines (IFN-γ, TNFα) and Th2-related cytokines (IL-4, IL-9) were relatively elevated in the remission phase compared to the acute phase, whereas the intensities of granulocyte-related cytokines (eotaxin, G-CSF, MCP-1, MIP-1b) were almost the same in the acute and remission phases of VKH disease patients. Therefore, we could hypothesize that the relative elevation of Th-related cytokines in the remission phase is an antagonistic response to the granulocyte-dominant immunity under GC therapy, and the antagonistic response implies potential readiness to induce relapse.

Concerning the effects of GC therapy on patients’ immunity, VKH disease patients in the remission phase were taking GC orally at daily doses of 2.5 to 5.0 mg. Although the GC doses are considerably low, even small amounts of GC could influence systemic immunity. A prospective long-term observational study of VKH disease patients not receiving systemic immunosuppressive therapy would be needed to understand the natural immune state in the remission phase of the disease.

As useful biomarkers for elucidating the underlying etiology of VKH disease, immune cells and their associated cytokines elevated both in the acute and remission phases could be appropriate candidates because they are less susceptible to influences due to disease stage and systemic immunosuppressive therapy.

In the hierarchical cluster analysis of leukocyte phenotypes (Figure 2), Cluster A (patterned red bar), consisting of basophils, effector memory CD4^+^ T cells, Th17-like, and plasmablasts, showed high intensity in the acute phase of VKH disease patients (brilliant green) compared to relative controls (brilliant red), and the intensity of the cluster in the remission phase (black to dark red) was also relatively high compared to relative controls. Furthermore, the cluster analysis of serum cytokine levels (Figure 4) indicated that the intensity of Cluster C (patterned yellow bar) consisting of IL-17A and IL-10 in the acute phase of VKH disease patients (brilliant green) was high compared to relative controls (brilliant red), and the intensity of the cluster in the remission phase (black) was also relatively high compared to relative controls. When focusing on the role of memory cells in the pathogenesis of VKH disease, the Th17 cell may be the most relevant candidate as a pathogenic lymphocyte.

The Th17 cell is not only involved in the clearance of extracellular pathogens during infections but also plays a role in the pathogenesis of several autoimmune and inflammatory diseases [43]. Th1 or Th17 cells are considered to be pathogenic in experimental autoimmune uveitis [44]. Currently, Th1 cells [5,6,26] and Th17 cells [6,7] are recognized as dominant pathogenic lymphocytes in VKH disease. Regarding the mechanism of cytokine-regulated Th cell differentiation, Th1 and Th17 cell subsets develop from the same naïve precursors expressing RAR-related orphan receptor C, T-bet, IL-23 receptor, type I IL-1 receptor, CD161, and chemokine receptor 6 [45]. These precursors coexist in the same microenvironment, in which IL-12 acts on established Th17 cells, driving the transition to Th1 lymphocytes through the intermediate phenotype of Th17/Th1 cells [45]. A recent hypothesis proposes that rather than Th17 cells, Th17-derived Th17/Th1 and Th1 cells play a critical role in the activities of autoimmune diseases, including insulin-dependent diabetes mellitus [46], autoimmune polyarthritis [47], and juvenile idiopathic arthritis [48], and are supported by the mechanism of differentiation and plasticity of Th17 cells [45]. Thus, the plasticity of Th17 cells toward the Th1 phenotype and the pathological roles of Th17, Th17/Th1, and Th1 cells may be particularly important in the debate of VKH disease pathology. In this regard, ustekinumab emerges as a promising molecular-targeted therapy for VKH disease [49]. Ustekinumab is a humanized monoclonal antibody that inhibits the binding of IL-12 and IL-23 to their receptors, consequently suppressing the differentiation of the naïve precursors into Th1, Th17/Th1, and Th17 cells. The drug is currently approved for moderate-to-severe psoriasis [50]. Future studies should explore Th17 cell-targeted therapy as a potentially effective treatment for refractory VKH disease.

Our study has several limitations. First, the sample size is not large enough to perform sub-analysis. VKH is a relatively rare disease with an annual prevalence and incidence that are estimated to be 15.5 and 6.5 per million, respectively, in Japan [51]. Therefore, our results may not reach statistical significance sufficiently, only indicating a trend of change. Second, the age in the VKH disease group is older than the relative control group, who are healthy volunteers recruited from the employees. Lazuardi et al. [52] analyzed the effects of age on T and B lymphocytes in human lymph nodes from young patients (mean age 11 years, range 1–20) in whom lymphadenectomy was performed for the routine diagnosis of cervical or axillar lymphadenopathy and elderly individuals (mean age 75 years, range 67–88) who underwent pelvic or cervical vascular reconstruction and reported that the relative number of CD8^+^ T cells decreases with age in human lymph nodes but the relative number of CD4^+^ T cells does not. So, the lower proportions of CD8^+^ T cells in the acute and remission phases of VKH disease patients compared to the relative controls may be due to their higher age and not VKH disease. Furthermore, it is well known that the proportion of naïve T cells in the peripheral blood is reduced with age. In addition, some of the other immune cell proportions may also be affected by age. In the future, a prospective intervention trial of systemic immunosuppressive therapy in VKH disease, which consists of age-matched controls and a larger sample size, will be needed to elucidate the complex inflammatory composition in the pathogenesis of VKH disease. Third, the follow-up period from primary onset to remission in VKH patients was approximately six months, and the patients in the remission phase were treated with low-dose GC. GC therapy could affect systemic immunity. Fourth, hierarchical cluster analysis is an unsupervised analysis, and some degrees of freedom are allowed in its interpretation.

## 5. Conclusions

Our study was designed to elucidate the comprehensive immune profiles of leukocytes and cytokines in the peripheral blood of VKH disease patients using mass cytometry and multiplex bead analysis. The property similarities among peripheral blood leukocytes and serum cytokines in VKH disease patients before and after GC therapy and in healthy relative controls were calculated by hierarchical cluster analysis. The inflammatory composition in peripheral blood during the acute phase of VKH disease did not show a distinct response derived from specific immune cells but exhibited part of complex hyperimmune responses induced primarily by Th cells and B lymphocytes, which resembled Type IV hypersensitivity reactions. Th17 cells and Th17-associated cytokines were relatively elevated throughout the acute and remission phases and may be major candidates for pathogenic immune cells in VKH disease.

## Figures and Tables

**Table 1:** Demographics and clinical data of VKH disease patients and controls.

Category												*p* Value	
*n*												VKH (Acute)	
Disease Stage		Acute			Remission				Remission			vs.	
		Median	First	Third	Median	First	Third		Median	First	Third	Control	VKH
			Quartile	Quartile		Quartile	Quartile			Quartile	Quartile		(Remission)
**Age**		66	60	70.5					30	26.5	31	0.038	
Gender (M/F)	1/2							4/3				1	
LogMAR VA		0	−0.04	0	0	−0.04	0		-			-	0.604
IOP		11.0	10.5	13.3	15.0	13.5	16.5		-			-	0.111
CFT		276.0	248.5	398.0	246.0	243.5	252.0		-			-	0.604
CRT		329.0	328.5	438.0	296.0	293.0	311.0		-			-	0.111
SFCT		194.0	185.0	206.5	227.0	163.5	232.5		-			-	1

VKH disease stage is classified into complete, incomplete, and probable based on ocular and laboratory findings [18]. Three VKH disease patients were diagnosed with incomplete VKH disease. Ocular symptoms in onset were bilateral in all of the patients. Data from the right eye were used for comparisons of ophthalmologic test values. VKH disease patients in the acute phase are the same as VKH disease patients in the remission phase. Age was compared between VKH disease and control groups by a Mann–Whitney *U* test. Gender was compared between the two groups by Fisher’s exact test. LogMAR VA, IOP, CFT, CRT, and SFCT were compared between the two groups by a Wilcoxon *t*-test. The *p*-value is two-tailed. CFT: central foveal thickness; CRT: central retinal thickness; F: female; IOP: intraocular pressure; logMAR: logarithm of the minimum angle of resolution; M: male; mmHg: millimeter of mercury; *n*: number; SFCT: subfoveal choroidal thickness; VA: visual acuity; VKH: Vogt–Koyanagi–Harada.

**Table 2:** Immune cell populations, phenotypes, and proportions among leukocytes in the peripheral blood of VKH disease patients.

Populations				Model Phenotypes		VKH					
*n*						3					
Disease Stage						Acute			Remission		
						Median	First	Third	Median	First	Third
							Quartile	Quartile		Quartile	Quartile
Intact live cells (%)											
Lymphocytes				CD3 T cells + B cells + NK cells + plasmablasts		61.1	53.6	70.8	41.5	25.3	45.3
	CD3^+^ T cells			CD8 T cells + CD4 T cells + γδ T cells + MAIT/NKT cells		36	32.2	37.8	22.7	14.6	27.9
		CD8^+^ T cells		CD3+ CD66b− CD19− CD8+ CD4− CD14− CD161− TCRγδ− CD123− CD11c−		4.98	4.25	6.27	3.14	2.35	3.79
			*Naïve*	CD8 T cells + CD45RA+ CCR7+ CD27+		1.95	1.73	2.13	0.75	0.59	1.22
			*Central memory*	CD8 T cells + CD45RA− CCR7+ CD27+		0.43	0.27	0.6	0.03	0.02	0.04
			*Effector memory*	CD8 T cells + CCR7− CD27+		0.8	0.78	2.24	1.46	0.95	1.58
			*Terminal effector*	CD8 T cells + CCR7− CD27−		1.18	0.98	1.5	0.7	0.68	0.98
		CD4^+^ T cells		CD66b− CD3+ CD8− CD4+ CD14− TCRγδ− CD11c−		25.7	24	29.9	18.2	11.3	22
			*Naïve*	CD4 T cells + CD45RA+ CCR7+ CD27+		14.7	13.3	19.9	10.2	6.16	12
			*Central memory*	CD4 T cells + CD45RA− CCR7+ CD27+		4.4	3.77	4.77	1.64	1.41	2.21
			*Effector memory*	CD4 T cells + CD45RA− CCR7− CD27+		4.41	3.83	5.13	4.2	2.46	5.28
			*Terminal effector*	CD4 T cells + CD45RA− CCR7− CD27−		1.3	1.05	1.67	2.12	1.28	2.54
			**Treg cells**	CD4 T cells + CD25+ CD127− CCR4+		0.66	0.51	0.7	0.12	0.12	0.36
			**Th1-like**	CD4 T cells + CXCR3+ CCR6− CXCR5− CCR4−		0.46	0.43	0.48	0.3	0.27	0.75
			**Th2-like**	CD4 T cells + CXCR3− CCR6− CXCR5− CCR4+		2.28	2.11	2.28	0.86	0.54	1.3
			**Th17-like**	CD4 T cells + CXCR3− CCR6+ CXCR5− CCR4+		2.2	1.72	2.69	2.19	1.28	2.43
		**γδ T cells**		CD66b− CD3+ CD8dim,− CD4− CD14− TCRγδ dim,+		1.03	0.81	1.22	0.41	0.36	1.08
		CD4^−^ T Cells									
			**MAIT/NKT cells**	CD66b− CD3+ CD4− CD14− CD161+ TCRγδ− CD28+ CD16−		1.1	0.89	1.19	0.98	0.58	1.01
	B cells			CD3− CD14− CD56− CD16 dim,− CD19+ CD20+ HLA-DR dim,+		12.9	9.09	22.9	7.76	4.44	9.01
		*Naïve*		B cells + CD27−		9.75	7.23	19.4	5.13	3.01	7.07
		*Memory*		B cells + CD27+		2.84	1.71	3.18	1	0.6	1.59
		*Plasmablasts*		CD3− CD14− CD16−,dim CD66b− CD20− CD19+ CD56− CD38++ CD27+		0.28	0.16	0.38	0.24	0.13	0.35
	NK cells			CD14− CD3− CD123− CD66b− CD45RA+ CD56 dim,+		12.3	10.01	12.3	5.83	3.7	8.44
		*Early*		NK cells + CD57−		2.85	2.08	4.41	1.73	1.35	2.76
		*Late*		NK cells + CD57+		6.33	5.6	8.67	4.11	2.35	5.68
Monocytes				CD3− CD19− CD56− CD66b− HLA-DR+ CD11c+		8.36	5.88	10.8	23.4	12.5	23.8
	**Classical**			Monocytes + CD14+ CD38+		7.77	5.48	9.78	22	11.7	22.7
	**Transitional**			Monocytes + CD14 dim CD38 dim		0.44	0.32	0.69	0.73	0.42	0.89
	**Nonclassical**			Monocytes + CD14− CD38−		0.15	0.09	0.37	0.14	0.11	0.25
Dendritic cells				pDCs+ mDCs		0.82	0.53	0.85	0.61	0.35	0.66
	**Plasmacytoid DCs**			CD3− CD19− CD14− CD20− CD66b− HLA-DR dim,+ CD11c− CD123+		0.25	0.15	0.26	0.06	0.03	0.1
	**Myeloid DCs**			CD3− CD19− CD14− CD20− HLA-DR dim,+ CD11c dim,+ CD123− CD16 dim,− CD38 dim,+ CD294− HLA−D		0.56	0.37	0.59	0.55	0.32	0.55
Granulocytes				Neutrophils + basophils + eosinophils + CD66b− neutrophils		9.81	7.32	11	15.7	14.8	50.9
	**Neutrophils**			CD66b dim,+ CD16+ HLA-DR−		7.6	5.62	8.46	13.4	12.1	49.5
	**Basophils**			HLA-DR− CD66b− CD123 dim,+ CD38+ CD294+		0.96	0.86	1.43	0.91	0.52	1.43
	**Eosinophils**			CD14− CD3− CD19− HLA-DR− CD294+ CD66b dim,+		0.38	0.25	0.42	0.11	0.1	0.17
	**CD66b^−^ neutrophils**			CD3− CD19− CD66b− CD56− HLA-DR− CD123− CD45−		0.53	0.32	0.77	0.88	0.59	1.09

Cell phenotypes are as defined by Bagwell et al. [22]. Nomenclature such as dim,+ means that dim to positive events are selected. Bold font denotes the classification of leukocytes based on differentiation and function. Italic font denotes the classification based on the maturity stage. CD: cluster of differentiation; DCs: dendritic cells; HLA: human leukocyte antigen; MAIT: mucosal-associated invariant T; mDCs: myeloid DCs; NK: natural killer; NKT: natural killer T; pDCs: plasmacytoid DCs; Th: T helper; Tregs: regulatory T cells.

**Table 3:** Immune cell populations, phenotypes, and proportions among leukocytes in the peripheral blood of controls and comparisons between VKH disease patients and controls.

Populations			Control			*p* Value		
*n*			7			Control vs.		VKH (Acute) vs.
			Median	First	Third	VKH	VKH	VKH
				Quartile	Quartile	(Acute)	(Remission)	(Remission)
Intact live cells (%)								
Lymphocytes			61.6	52.0	75.6	0.595	0.082	0.111
CD3^+^ T cells			43.7	35.9	48.8	0.242	0.082	0.111
	CD8^+^ T cells		14.6	9.99	15.8	**0.038**	**0.038**	0.111
		*Naïve*	7.26	5.44	9.90	0.082	0.056	0.111
		*Central memory*	0.20	0.12	0.25	0.385	**0.038**	0.111
		*Effector memory*	1.83	1.59	2.88	0.445	0.175	0.604
		*Terminal effector*	3.33	2.09	4.44	0.175	0.082	0.287
	CD4^+^ T cells		23.2	20.5	27.0	0.521	0.242	0.287
		*Naïve*	14.2	12.5	16.5	0.558	0.175	0.111
		*Central memory*	2.09	1.83	2.66	0.082	0.445	0.111
		*Effector memory*	4.04	1.55	5.32	0.558	0.595	1.000
		*Terminal effector*	1.90	1.64	2.81	0.242	0.595	0.287
		**Treg cells**	0.47	0.42	0.81	0.595	0.175	0.111
		**Th1-like**	1.00	0.30	1.27	0.332	0.539	1
		**Th2-like**	1.36	1.26	1.71	0.123	0.332	0.111
		**Th17-like**	1.25	1.11	1.43	0.175	0.445	0.604
	**γδ** **T cells**		2.32	2.03	3.10	0.056	0.056	0.604
	CD4^−^ T Cells							
		**MAIT/NKT cells**	0.92	0.59	2.71	0.595	0.558	0.111
	B cells		10.5	6.51	11.3	0.445	0.332	0.111
	*Naïve*		7.40	4.88	8.90	0.332	0.445	0.111
	*Memory*		2.08	1.57	2.71	0.521	0.332	0.111
	*Plasmablasts*		0.06	0.06	0.07	0.445	0.521	0.111
	NK cells		15.7	7.64	18.1	0.521	0.242	0.287
	*Early*		5.70	1.99	6.46	0.445	0.242	0.604
	*Late*		8.89	5.66	10.7	0.558	0.175	0.604
Monocytes			9.65	7.29	10.0	0.558	0.445	0.287
**Classical**			8.55	6.20	8.68	0.595	0.445	0.287
**Transitional**			0.82	0.75	1.13	0.242	0.332	1
**Nonclassical**			0.48	0.22	0.56	0.332	0.175	1
Dendritic cells			0.79	0.57	0.93	0.558	0.242	0.111
**Plasmacytoid DCs**			0.12	0.08	0.19	0.503	0.385	0.111
**Myeloid DCs**			0.65	0.49	0.70	0.332	0.332	0.111
Granulocytes			17.0	5.82	25.9	0.521	0.521	0.111
**Neutrophils**			16.4	3.63	23.6	0.521	0.521	0.111
**Basophils**			1.08	0.48	1.29	0.595	0.613	0.604
**Eosinophils**			0.04	0.03	0.56	0.445	0.558	0.111
**CD66b^−^ neutrophils**			0.57	0.41	0.67	0.558	0.445	0.604

Comparisons between VKH disease and control groups were evaluated by a Mann–Whitney *U* test. Comparisons between acute and remission phases in the VKH disease groups were performed by a Mann–Whitney *U* test. The *p*-value is two-tailed.

**Table 4:** Serum cytokine levels in VKH disease patients and controls.

Category	VKH								Control			
*n*	3								7			
Disease Stage	Acute				Remission							
	Detectable	Median	First	Third	Detectable	Median	First	Third	Detectable	Median	First	Third
	Rate (%)		Quartile	Quartile	Rate (%)		Quartile	Quartile	Rate (%)		Quartile	Quartile
PDGF-BB	3 (100)	345.4	232.1	800.5	3 (100)	288.4	171.2	760.2	7 (100)	360.4	127.7	810.1
IL-1β	2 (66.7)	0.39	0.20	0.47	0 (0)	0	0	0	4 (57.1)	0.31	0	0.58
IL-1ra	3 (100)	24.0	22.8	30.0	3 (100)	36.1	33.6	39.1	4 (57.1)	13.1	0	32.1
IL-2	0 (0)	0	0	0	0 (0)	0	0	0	0 (0)	0	0	0
**IL-4**	3 (100)	1.63	1.43	2.90	3 (100)	3.46	3.45	4.08	5 (71.4)	1.36	0.14	2.05
IL-5	0 (0)	0	0	0	0 (0)	0	0	0	0 (0)	0	0	0
IL-6	0 (0)	0	0	0	0 (0)	0	0	0	0 (0)	0	0	0
IL-7	2 (66.7)	33.7	16.8	42.7	1 (33.3)	0	0	12.8	3 (42.9)	0	0	17.5
IL-8	1 (33.3)	0	0	1.28	3 (100)	1.50	1.40	2.16	2 (28.6)	0	0	0.55
IL-9	3 (100)	27.4	24.1	38.3	3 (100)	44.4	43.8	45.8	4 (57.1)	2.89	0	40.7
IL-10	1 (33.3)	0	0	19.9	1 (33.3)	0	0	9.22	0 (0)	0	0	0
IL-12	1 (33.3)	0	0	12.5	0 (0)	0	0	0	0 (0)	0	0	0
IL-13	1 (33.3)	0	0	2.18	0 (0)	0	0	0	0 (0)	0	0	0
IL-15	0 (0)	0	0	0	0 (0)	0	0	0	0 (0)	0	0	0
IL-17A	2 (66.7)	3.38	1.69	4.29	2 (66.7)	3.05	1.53	3.63	2 (28.6)	0	0	2.96
Eotaxin	3 (100)	139.6	119.0	168.1	3 (100)	168.8	94.1	180.2	7 (100)	9.29	6.07	84.0
bFGF	2 (66.7)	4.94	2.47	5.47	0 (0)	0	0	0	4 (57.1)	4.67	0	4.94
G-CSF	2 (66.7)	142.8	71.4	331.3	3 (100)	166.5	132.9	264.2	1 (14.3)	0	0	0
GM-CSF	0 (0)	0	0	0	0 (0)	0	0	0	0 (0)	0	0	0
IFN-γ	0 (0)	0	0	0	1 (33.3)	0	0	0.88	0 (0)	0	0	0
IP-10	3 (100)	542.8	347.9	695.0	3 (100)	70.5	61.4	101.7	7 (100)	63.9	50.6	225.0
**MCP-1**	3 (100)	5.10	4.68	6.78	3 (100)	7.16	6.48	7.97	2 (28.6)	0	0	0.30
MIP-1α	2 (66.7)	0.19	0.10	0.69	3 (100)	0.53	0.49	1.45	3 (42.9)	0	0	0.72
MIP-1β	3 (100)	26.3	24.4	26.7	3 (100)	23.0	20.9	29.8	5 (71.4)	5.78	1.71	32.8
RANTES	3 (100)	937.3	842.9	973.7	3 (100)	580.3	395.5	614.8	7 (100)	93.5	56.9	333.9
TNFα	2 (66.7)	7.32	3.66	11.4	2 (66.7)	13.4	6.68	17.3	2 (28.6)	0	0	7.74
VEGF-A	0 (0)	0	0	0	0 (0)	0	0	0	0 (0)	0	0	0

bFGF: basic fibroblast growth factor; G-CSF: granulocyte colony-stimulating factor; GM-CSF: granulocyte macrophage colony-stimulating factor; IFN: interferon; IP-10: interferon gamma-induced protein 10; IL: interleukin; MIP: macrophage inflammatory protein; MCP: monocyte chemotactic protein; PDGF: platelet-derived growth factor; ra: receptor antagonist; RANTES: regulated on activation; normal T cell expressed and secreted; TNF: tumor necrosis factor; VEGF: vascular endothelial growth factor. Cytokine levels are expressed in pg/mL.

**Table 5:** Comparisons of serum cytokine levels in VKH disease patients and controls.

Category	*p* Value			Detection Range		
	Control vs.		VKH (Acute) vs.			
	VKH	VKH	VKH			
	(Acute)	(Remission)	(Remission)			
PDGF-BB	0.595	0.595	0.111	7.67	to	42,619.2
IL-1β	0.613	0.202	0.183	0.28	to	5036.8
IL-1ra	0.445	0.242	0.183	8.56	to	37,276.7
IL-2	0.613	0.613	1	1.50	to	8778.3
**IL-4**	0.445	**0.038**	0.111	0.16	to	3539.9
IL-5	0.613	0.613	1	4.02	to	85,349.0
IL-6	0.613	0.613	1	0.37	to	5969.6
IL-7	0.242	0.595	0.183	1.84	to	36,229.8
IL-8	0.595	0.123	0.111	0.51	to	10,416.3
IL-9	0.332	0.175	0.287	0.92	to	21,827.0
IL-10	0.445	0.445	0.319	0.84	to	12,755.7
IL-12	0.445	0.613	0.319	1.58	to	21,263.1
IL-13	0.445	0.613	0.319	0.75	to	5003.2
IL-15	0.613	0.613	1	231.8	to	76,422.6
IL-17A	0.539	0.539	0.604	2.65	to	35,294.2
Eotaxin	0.123	0.123	0.604	0.09	to	1487.0
bFGF	0.539	0.202	0.183	3.52	to	5445.8
G-CSF	0.175	0.056	1	55.0	to	70,102.6
GM-CSF	0.613	0.613	1	0.33	to	1822.9
IFN-γ	0.613	0.445	0.319	0.74	to	22,825.9
IP-10	0.123	0.595	0.111	1.53	to	23,765.1
**MCP-1**	**0.038**	**0.038**	0.287	0.34	to	5761.9
MIP-1α	0.558	0.242	0.111	0.05	to	348.6
MIP-1β	0.558	0.445	0.604	0.46	to	2126.1
RANTES	0.123	0.175	0.111	1.00	to	5544.2
TNFα	0.521	0.503	0.183	2.73	to	53,796.5
VEGF-A	0.613	0.613	1	19.5	to	69,174.0

Comparisons between VKH disease and control groups were evaluated by a Mann–Whitney *U* test. Comparisons between acute and remission phases in the VKH disease groups were performed by a Mann–Whitney *U* test. The *p*-value is two-tailed.

## Data Availability

The original contributions presented in the study are included in the article/Supplementary Materials. Further inquiries can be directed to the corresponding author.

## Supplementary Materials

The following supporting information can be downloaded at https://www.mdpi.com/article/10.3390/jcm12247742/s1, Figure S1: Clinical course of No. 1 VKH disease patient under glucocorticoid therapy; Figure S2: Clinical course of No. 2 VKH disease patient under glucocorticoid therapy; Figure S3: Clinical course of No. 3 VKH disease patient under glucocorticoid therapy; Table S1: Immune cell populations, phenotypes and proportions among leukocytes in peripheral blood of each VKH disease patient; Table S2: Immune cell populations, phenotypes and proportions among leukocytes in peripheral blood of each healthy control; Table S3: Hematologic data of No. 1 VKH disease patient; Table S4: Hematologic data of No. 2 VKH disease patient; Table S5: Hematologic data of No. 3 VKH disease patient; Table S6: Serum cytokine levels in each VKH disease patient; Table S7: Serum cytokine levels in each control.

## Abbreviations

CD; cluster of differentiation; CFT: central foveal thickness; CRT: central retinal thickness; DCs: dendritic cells; GC: glucocorticoid; G-CSF: granulocyte colony-stimulating factor; γδ: gamma delta; IFN-γ: interferon-gamma; IL: interleukin; IP-10: interferon gamma-inducible protein 10; logMAR: logarithm of the minimum angle of resolution; MAIT: mucosal-associated invariant T; MCP-1: monocyte chemotactic protein-1; mDCs: myeloid DCs; MIP: macrophage inflammatory protein; NK: natural killer; NKT: natural killer T; pDCs: plasmacytoid DCs; ra: receptor antagonist; RANTES: regulated on activation; normal T cell expressed and secreted; SFCT: subfoveal choroidal thickness; Th1: T helper type 1; Th2: T helper type 2; Th17: T helper type 17; TNFα: tumor necrosis factor α; Tregs: regulatory T cells; VEGF: vascular endothelial growth factor; VKH: Vogt–Koyanagi–Harada.

## References

[1] Chang J.H.M., Wakefield D. (2002). Uveitis: A global perspective. Ocul. Immunol. Inflamm..

[2] Suttorp-Schulten M.S., Rothova A. (1996). The possible impact of uveitis in blindness: A literature survey. Br. J. Ophthalmol..

[3] Abu El-Asrar A.M., Van Damme J., Struyf S., Opdenakker G. (2021). New Perspectives on the Immunopathogenesis and Treatment of Uveitis Associated With Vogt-Koyanagi-Harada Disease. Front. Med..

[4] Maezawa N., Yano A., Taniguchi M., Kojima S. (1982). The role of cytotoxic T lymphocytes in the pathogenesis of Vogt-Koyanagi-Harada disease. Ophthalmologica.

[5] Herbort C.P., Mochizuki M. (2007). Vogt–Koyanagi–Harada disease: Inquiry into the genesis of a disease name in the historical context of Switzerland and Japan. Int. Ophthalmol..

[6] Wu S., Ma R., Zhong Y., Chen Z., Zhou H., Zhou M., Chong W., Chen J. (2021). Deficiency of IL-27 Signaling Exacerbates Experimental Autoimmune Uveitis with Elevated Uveitogenic Th1 and Th17 Responses. Int. J. Mol. Sci..

[7] Chi W., Yang P., Li B., Wu C., Jin H., Zhu X., Chen L., Zhou H., Huang X., Kijlstra A. (2007). IL-23 promotes CD4^+^ T cells to produce IL-17 in Vogt-Koyanagi-Harada disease. J. Allergy Clin. Immunol..

[8] Sakamoto T., Murata T., Inomata H. (1991). Class II major histocompatibility complex on melanocytes of Vogt-Koyanagi-Harada disease. Arch. Ophthalmol..

[9] Jiang H., Li Z., Yu L., Zhang Y., Zhou L., Wu J., Yuan J., Han M., Xu T., He J. (2021). Immune Phenotyping of Patients With Acute Vogt-Koyanagi-Harada Syndrome Before and After Glucocorticoids Therapy. Front. Immunol..

[10] Jakubzick C.V., Randolph G.J., Henson P.M. (2017). Monocyte differentiation and antigen-presenting functions. Nat. Rev. Immunol..

[11] Ziegler-Heitbrock L., Ancuta P., Crowe S., Dalod M., Grau V., Hart D.N., Leenen P.J.M., Liu Y.-J., MacPherson G., Randolph G.J. (2010). Nomenclature of monocytes and dendritic cells in blood. Blood.

[12] Cros J., Cagnard N., Woollard K., Patey N., Zhang S.Y., Senechal B., Puel A., Biswas S.K., Moshous D., Picard C. (2010). Human CD14dim monocytes patrol and sense nucleic acids and viruses via TLR7 and TLR8 receptors. Immunity.

[13] Geissmann F., Jung S., Littman D.R. (2003). Blood monocytes consist of two principal subsets with distinct migratory properties. Immunity.

[14] Weber C., Belge K.U., Von Hundelshausen P., Draude G., Steppich B., Mack M., Frankenberger M., Weber K.S., Ziegler-Heitbrock H.W. (2000). Differential chemokine receptor expression and function in human monocyte subpopulations. J. Leukoc. Biol..

[15] Ancuta P., Rao R., Moses A., Mehle A., Shaw S.K., Luscinskas F.W., Gabuzda D. (2003). Fractalkine preferentially mediates arrest and migration of CD16^+^ monocytes. J. Exp. Med..

[16] Wong K.L., Tai J.J.-Y., Wong W.-C., Han H., Sem X., Yeap W.-H., Kourilsky P., Wong S.-C. (2011). Gene expression profiling reveals the defining features of the classical, intermediate, and nonclassical human monocyte subsets. Blood.

[17] Kapellos T.S., Bonaguro L., Gemünd I., Reusch N., Saglam A., Hinkley E.R., Schultze J.L. (2019). Human monocyte subsets and phenotypes in major chronic inflammatory diseases. Front. Immunol..

[18] Read R.W., Holland G.N., Rao N.A., Tabbara K.F., Ohno S., Arellanes-Garcia L., Pivetti-Pezzi P., Tessler H.H., Usui M. (2001). Revised diagnostic criteria for Vogt-Koyanagi-Harada disease: Report of an international committee on nomenclature. Am. J. Ophthalmol..

[19] Chui T.Y., VanNasdale D.A., Elsner A.E., Burns S.A. (2014). The association between the foveal avascular zone and retinal thickness. Investig. Ophthalmol. Vis. Sci..

[20] Group ETDRSR (1987). Photocoagulation for diabetic macular edema: Early Treatment Diabetic Retinopathy Study report no. 4. Int. Ophthalmol. Clin..

[21] Branchini L.A., Adhi M., Regatieri C.V., Nandakumar N., Liu J.J., Laver N., James G Fujimoto J.G., Duker J.S. (2013). Analysis of choroidal morphologic features and vasculature in healthy eyes using spectral-domain optical coherence tomography. Ophthalmology.

[22] Bagwell C.B., Hunsberger B., Hill B., Herbert D., Bray C., Selvanantham T., Li S., Villasboas J.C., Pavelko K., Strausbauch M. (2020). Multi-site reproducibility of a human immunophenotyping assay in whole blood and peripheral blood mononuclear cells preparations using CyTOF technology coupled with Maxpar Pathsetter, an automated data analysis system. Cytometry B Clin. Cytom..

[23] Sato T., Enoki T., Karasawa Y., Someya H., Taguchi M., Harimoto K., Takayama K., Kanda T., Ito M., Takeuchi M. (2021). Inflammatory Factors of Macular Atrophy in Eyes with Neovascular Age-Related Macular Degeneration Treated with Aflibercept. Front. Immunol..

[24] Cohen J. (1992). A power primer. Psychol. Bull..

[25] Wilks D.S. (2011). Cluster analysis. International Geophysics. 100.

[26] Yamaki K., Gocho K., Hayakawa K., Kondo I., Sakuragi S. (2000). Tyrosinase family proteins are antigens specific to Vogt-Koyanagi-Harada disease. J. Immunol..

[27] Liang L., Peng X.-Y., Wang H. (2019). Th lymphocyte subsets in patients with Vogt-Koyanagi-Harada disease. Int. J. Ophthalmol..

[28] Opdenakker G., El-Asrar A.A., Van Damme J. (2020). Remnant epitopes generating autoimmunity: From model to useful paradigm. Trends Immunol..

[29] Rajan T.V. (2003). The Gell-Coombs classification of hypersensitivity reactions: A re-interpretation. Trends Immunol..

[30] Gell P.G.H., Coombs R.R.A. (1963). Clinical Aspects of Immunology.

[31] Bendiner E. (1981). Baron von Pirquet: The aristocrat who discovered and defined allergy. Hosp. Pract. (Off. Ed.).

[32] Inomata H., Sakamoto T. (1990). Immunohistochemical studies of Vogt-Koyanagi-Harada disease with sunset sky fundus. Curr. Eye Res..

[33] Damico F.M., Cunha-Neto E., Goldberg A.C., Iwai L.K., Marin M.L., Hammer J., Jorge Kalil J., Yamamoto J.H. (2005). T-cell recognition and cytokine profile induced by melanocyte epitopes in patients with HLA-DRB1*0405-positive and -negative Vogt-Koyanagi-Harada uveitis. Investig. Ophthalmol. Vis. Sci..

[34] Hammer H. (1974). Cellular hypersensitivity to uveal pigment confirmed by leucocyte migration tests in sympathetic ophthalmitis and the Vogt-Koyanagi-Harada syndrome. Br. J. Ophthalmol..

[35] Kay A.B. (2001). Allergy and allergic diseases. N. Engl. J. Med..

[36] Goto H., Zako M., Namba K., Hashida N., Kaburaki T., Miyazaki M., Sonoda K.-H., Abe T., Mizuki N., Kamoi K. (2019). Adalimumab in active and inactive, non-infectious uveitis: Global results from the VISUAL I and VISUAL II trials. Ocul. Immunol. Inflamm..

[37] Liu X., Jiang Q., Lv J., Yang S., Huang Z., Duan R., Tao T., Li Z., Ju R., Zheng Y. (2022). Insights gained from single-cell analysis of immune cells in tofacitinib treatment of Vogt-Koyanagi-Harada disease. JCI Insight..

[38] Gerlach K., Lechner K., Popp V., Offensperger L., Zundler S., Wiendl M., Becker E., Atreya R., Rath T., Neurath M.F. (2021). The JAK1/3 inhibitor to tofacitinib suppresses T cell homing and activation in chronic intestinal inflammation. J. Crohn’s Colitis.

[39] Papasavvas I., Tugal-Tutkun I., Herbort C.P. (2020). Vogt–Koyanagi–Harada is a curable autoimmune disease: Early diagnosis and immediate dual steroidal and non-steroidal immunosuppression are crucial prerequisites. J. Curr. Ophthalmol..

[40] Read R.W., Rechodouni A., Butani N., Johnston R., LaBree L.D., Smith R.E., Rao N.A. (2001). Complications and prognostic factors in Vogt-Koyanagi-Harada disease. Am. J. Ophthalmol..

[41] Meijsing S.H. (2015). Mechanisms of Glucocorticoid-Regulated Gene Transcription. Adv. Exp. Med. Biol..

[42] Sloka J., Stefanelli M. (2005). The mechanism of action of methylprednisolone in the treatment of multiple sclerosis. Mult. Scler..

[43] Oukka M. (2008). Th17 cells in immunity and autoimmunity. Ann. Rheum. Dis..

[44] Luger D., Silver P.B., Tang J., Cua D., Chen Z., Iwakura Y., Bowman E.D., Sgambellone N.M., Chan C.-C., Caspi R.R. (2008). Either a Th17 or a Th1 effector response can drive autoimmunity: Conditions of disease induction affect dominant effector category. J. Exp. Med..

[45] Cosmi L., Liotta F., Maggi E., Romagnani S., Annunziato F. (2011). Th17 cells: New players in asthma pathogenesis. Allergy.

[46] Bending D., De la Peña H., Veldhoen M., Phillips J.M., Uyttenhove C., Stockinger B., Cooke A. (2009). Highly purified Th17 cells from BDC2.5NOD mice convert into Th1-like cells in NOD/SCID recipient mice. J. Clin. Investig..

[47] Nistala K., Adams S., Cambrook H., Ursu S., Olivito B., de Jager W., Evans J.G., Cimaz R., Bajaj-Elliott M., Wedderburn L.R. (2010). Th17 plasticity in human autoimmune arthritis is driven by the inflammatory environment. Proc. Natl. Acad. Sci. USA.

[48] Cosmi L., Cimaz R., Maggi L., Santarlasci V., Capone M., Borriello F., Frosali F., Querci V., Simonini G., Barra G. (2011). CD4^+^ CD161^+^ T cells showing transient nature of the Th17 phenotype are present in the synovial fluid from patients with juvenile idiopathic arthritis. Arthritis Rheum..

[49] Baker K.F., Isaacs J.D. (2018). Novel therapies for immune-mediated inflammatory diseases: What can we learn from their use in rheumatoid arthritis, spondyloarthritis, systemic lupus erythematosus, psoriasis, Crohn’s disease and ulcerative colitis?. Ann. Rheum. Dis..

[50] Leonardi C.L., Kimball A.B., Papp K.A., Yeilding N., Guzzo C., Wang Y., Li S., Dooley L.T., Gordon K.B. (2008). Efficacy and safety of ustekinumab, a human interleukin-12/23 monoclonal antibody, in patients with psoriasis: 76-week results from a randomised, double-blind, placebo-controlled trial (PHOENIX 1). Lancet.

[51] Murakami S., Inaba Y., Mochizuki M., Nakajima A., Urayama A. (1994). A nationwide survey on the occurrence of Vogt-Koyanagi-Harada disease in Japan. Jpn. J. Ophthalmol..

[52] Lazuardi L., Jenewein B., Wolf A.M., Pfister G., Tzankov A., Grubeck-Loebenstein B. (2005). Age-related loss of naive T cells and dysregulation of T-cell/B-cell interactions in human lymph nodes. Immunology.

