# Supplementary material for: Classification of Peripheral Blood Leukocyte Phenotypes and Serum Cytokines in Vogt–Koyanagi–Harada Disease before and after Glucocorticoid Therapy
Source: J Clin Med. 2023 Dec 17;12(24):7742. doi: 10.3390/jcm12247742 (PMC10743729; doi:10.3390/jcm12247742)
Supplement: Supplementary file 1 [file jcm-12-07742-s001.zip › jcm-2765368-supplementary.pdf]

Journal of
Clinical Medicine

MDPI

Table S1. Immune cell populations, phenotypes and proportions among leukocytes in peripheral blood of each VKH disease patient.

Populations	Model Phenotypes	VKH					
Disease Stage		Acute			Remission		
Patient Number		No.1	No.2	No.3	No.1	No.2	No.3
Intact live cells (%)		100	100	100	100	100	100
Lymphocytes	CD3 T cells + B cells + NK cells + plasmablasts	61.1	80.4	46.1	41.5	49.2	9.14
CD3^{+} T cells	CD8 T cells + CD4 T cells + γδ T cells + MAIT/NKT cells	36.0	39.7	28.5	22.7	33.1	6.48
CD8^{+} T cells	CD3+ CD66b- CD19- CD8+ CD4- CD14- CD161- TCRγδ- CD123- CD11c-	7.55	3.51	4.98	3.14	4.43	1.56
Naïve	CD8 T cells + CD45RA+ CCR7+ CD27+	1.95	1.51	2.31	0.75	1.68	0.42
Central memory	CD8 T cells + CD45RA- CCR7+ CD27+	0.76	0.43	0.10	0.03	0.04	0.01
Effector memory	CD8 T cells + CCR7- CD27+	3.67	0.80	0.76	1.70	1.46	0.43
Terminal effector	CD8 T cells + CCR7- CD27-	1.18	0.77	1.81	0.66	1.26	0.70
CD4^{+} T cells	CD66b- CD3+ CD8- CD4+ CD14- TCRγδ- CD11c-	25.7	34.1	22.3	18.2	25.9	4.44
Naïve	CD4 T cells + CD45RA+ CCR7+ CD27+	14.7	25.0	11.9	10.2	13.8	2.12
Central memory	CD4 T cells + CD45RA- CCR7+ CD27+	3.13	4.40	5.14	1.64	2.77	1.17
Effector memory	CD4 T cells + CD45RA- CCR7- CD27+	5.85	3.24	4.41	4.20	6.35	0.72
Terminal effector	CD4 T cells + CD45RA- CCR7- CD27-	2.03	1.30	0.80	2.12	2.95	0.43
**Treg cells**	CD4 T cells + CD25+ CD127- CCR4+	0.73	0.66	0.36	0.12	0.59	0.12
**Th1-like**	CD4 T cells + CXCR3+ CCR6- CXCR5- CCR4-	0.39	0.50	0.46	0.30	1.19	0.24
**Th2-like**	CD4 T cells + CXCR3- CCR6- CXCR5- CCR4+	2.28	2.28	1.94	0.86	1.73	0.21
**Th17-like**	CD4 T cells + CXCR3- CCR6+ CXCR5- CCR4+	3.17	1.24	2.20	2.66	2.19	0.36
**γδ T cells**	CD66b- CD3+ CD8dim,- CD4- CD14- TCRγδ dim,+	1.40	1.03	0.59	0.41	1.75	0.30
CD4^{-} T Cells							
**MAIT/NKT cells**	CD66b- CD3+ CD4- CD14- CD161+ TCRγδ- CD28+ CD16-	1.28	1.10	0.67	0.98	1.04	0.18
B cells	CD3- CD14- CD56- CD16 dim,- CD19+ CD20+ HLA-DR dim,+	12.9	33.0	5.30	7.76	10.3	1.11
Naïve	B cells + CD27-	9.75	28.99	4.71	5.13	9.00	0.88
Memory	B cells + CD27+	2.84	3.51	0.57	2.18	1.00	0.20
Plasmablasts	CD3- CD14- CD16-,dim CD66b- CD20- CD19+ CD56- CD38++ CD27+	0.28	0.48	0.03	0.45	0.24	0.02
NK cells	CD14- CD3- CD123- CD66b- CD45RA+ CD56 dim,+	12.3	7.72	12.3	11.0	5.83	1.56
Early	NK cells + CD57-	5.97	2.85	1.31	3.79	1.73	0.97
Late	NK cells + CD57+	6.33	4.87	11.0	7.25	4.11	0.59
Monocytes	CD3- CD19- CD56- CD66b- HLA-DR+ CD11c+	13.3	8.36	3.40	23.4	24.1	1.55
**Classical**	Monocytes + CD14+ CD38+	11.8	7.77	3.19	22.0	23.3	1.31
**Transitional**	Monocytes + CD14 dim CD38 dim	0.94	0.44	0.19	1.05	0.73	0.10
**Nonclassical**	Monocytes + CD14- CD38-	0.58	0.15	0.02	0.35	0.07	0.14
Dendritic cells	pDCs+ mDCs	0.88	0.82	0.23	0.61	0.70	0.09
**Plasmacytoid DCs**	CD3- CD19- CD14- CD20- CD66b- HLA-DR dim,+ CD11c- CD123+	0.25	0.26	0.05	0.06	0.14	0.00
**Myeloid DCs**	CD3- CD19- CD14- CD20- HLA-DR dim,+ CD11c dim,+ CD123- CD16 dim,- CD38 dim,+ CD294- HLA-D	0.62	0.56	0.18	0.55	0.55	0.09
Granulocytes	Neutrophils + basophils + eosinophils + CD66b- neutrophils	12.1	4.82	9.81	15.7	13.9	86.1
**Neutrophils**	CD66b dim,+ CD16+ HLA-DR-	9.31	3.64	7.60	13.4	10.8	85.6
**Basophils**	HLA-DR- CD66b- CD123 dim,+ CD38+ CD294+	1.89	0.96	0.75	0.9	1.94	0.13
**Eosinophils**	CD14- CD3- CD19- HLA-DR- CD294+ CD66b dim,+	0.38	0.11	0.46	0.08	0.22	0.11
**CD66b^{-} neutrophils**	CD3- CD19- CD66b- CD56- HLA-DR- CD123- CD45-	0.53	0.11	1.00	1.30	0.88	0.30

Cell phenotypes are as defined by Bagwell et al [22]. Nomenclature such as dim,+ means that dim to positive events are selected. Bold font denotes classification of leukocytes based on differentiation and function. Italic font denotes the classification based on maturity stage. CD; cluster of differentiation, DCs; dendritic cells, HLA; human leukocyte antigen, MAIT; mucosal associated invariant T,

mDCs; myeloid DCs, NK; natural killer, NKT; natural killer T, pDCs; plasmacytoid DCs, Th; T helper, Tregs; regulatory T cells, VKH; Vogt-Koyanagi-Harada.

Table S2. Immune cell populations, phenotypes and proportions among leukocytes in peripheral blood of each healthy control.

Populations	Model Phenotypes	Controls						
**Reference Number**		**No.1**	**No.2**	**No.3**	**No.4**	**No.5**	**No.6**	**No.7**
Intact live cells (%)		100	100	100	100	100	100	100
Lymphocytes	CD3 T cells + B cells + NK cells + plasmablasts	46.8	45.1	80.8	78.3	61.6	72.8	57.2
$CD3^+$ T cells	CD8 T cells + CD4 T cells + γδ T cells + MAIT/NKT cells	28.6	23.3	50.2	43.3	47.4	43.7	50.6
$CD8^+$ T cells	CD3+ CD66b- CD19- CD8+ CD4- CD14- CD161- TCRγδ- CD123- CD11c-	8.14	8.69	11.3	16.6	14.6	15.0	19.0
Naïve	CD8 T cells + CD45RA+ CCR7+ CD27+	1.62	5.96	4.92	9.02	7.26	10.77	14.59
Central memory	CD8 T cells + CD45RA- CCR7+ CD27+	0.10	0.20	0.33	0.24	0.13	0.26	0.11
Effector memory	CD8 T cells + CCR7- CD27+	1.83	1.57	2.69	3.06	1.60	3.07	1.12
Terminal effector	CD8 T cells + CCR7- CD27-	4.59	0.97	3.33	4.29	5.61	0.95	3.21
$CD4^+$ T cells	CD66b- CD3+ CD8- CD4+ CD14- TCRγδ- CD11c-	18.4	11.9	32.0	23.2	26.2	22.7	27.7
Naïve	CD4 T cells + CD45RA+ CCR7+ CD27+	12.9	6.54	18.2	14.2	14.9	12.0	21.1
Central memory	CD4 T cells + CD45RA- CCR7+ CD27+	2.09	1.95	4.86	1.70	2.62	2.70	1.18
Effector memory	CD4 T cells + CD45RA- CCR7- CD27+	1.56	1.54	6.19	4.44	0.0	6.95	4.04
Terminal effector	CD4 T cells + CD45RA- CCR7- CD27-	1.84	1.90	2.77	2.85	8.91	1.02	1.43
**Treg cells**	CD4 T cells + CD25+ CD127- CCR4+	0.47	0.30	0.97	0.69	0.44	0.93	0.40
**Th1-like**	CD4 T cells + CXCR3+ CCR6- CXCR5- CCR4-	0.04	0.07	1.50	1.00	1.35	1.19	0.52
**Th2-like**	CD4 T cells + CXCR3- CCR6- CXCR5- CCR4+	1.33	1.36	1.72	1.19	2.83	1.08	1.69
**Th17-like**	CD4 T cells + CXCR3- CCR6+ CXCR5- CCR4+	1.22	1.00	2.06	1.25	0.68	1.43	1.43
**γδ T cells**	CD66b- CD3+ CD8dim,- CD4- CD14- TCRγδ dim,+	1.10	2.32	3.16	1.78	6.51	2.27	3.03
$CD4^-$ T Cells								
**MAIT/NKT cells**	CD66b- CD3+ CD4- CD14- CD161+ TCRγδ- CD28+ CD16-	0.92	0.39	3.80	1.72	0	3.69	0.79
B cells	CD3- CD14- CD56- CD16 dim,- CD19+ CD20+ HLA-DR dim,+	7.52	5.50	10.5	10.7	11.8	13.5	2.05
Naïve	B cells + CD27-	5.64	4.12	7.40	8.42	9.37	10.30	1.86
Memory	B cells + CD27+	1.82	1.32	3.04	2.08	2.38	3.11	0.15
Plasmablasts	CD3- CD14- CD16-,dim CD66b- CD20- CD19+ CD56- CD38++ CD27+	0.06	0.06	0.06	0.25	0.07	0.05	0.04
NK cells	CD14- CD3- CD123- CD66b- CD45RA+ CD56 dim,+	10.7	16.2	20.1	24.3	2.43	15.7	4.60
Early	NK cells + CD57-	2.36	6.13	9.78	5.70	1.52	6.78	1.61
Late	NK cells + CD57+	8.31	10.1	11.3	18.6	0.91	8.89	3.00
Monocytes	CD3- CD19- CD56- CD66b- HLA-DR+ CD11c+	5.78	10.2	6.15	9.88	9.65	11.1	8.42
**Classical**	Monocytes + CD14+ CD38+	4.29	8.59	5.61	9.02	8.55	8.77	6.79
**Transitional**	Monocytes + CD14 dim CD38 dim	0.81	1.12	0.43	0.69	0.82	1.69	1.14
**Nonclassical**	Monocytes + CD14- CD38-	0.68	0.48	0.11	0.17	0.27	0.63	0.48
Dendritic cells	pDCs+ mDCs	0.63	0.85	0.79	1.00	0.48	1.08	0.50
**Plasmacytoid DCs**	CD3- CD19- CD14- CD20- CD66b- HLA-DR dim,+ CD11c- CD123+	0.12	0.10	0.14	0.35	0.03	0.24	0.05
**Myeloid DCs**	CD3- CD19- CD14- CD20- HLA-DR dim,+ CD11c dim,+ CD123- CD16 dim,- CD38 dim,+ CD294- HLA-D	0.51	0.75	0.65	0.65	0.45	0.84	0.46
Granulocytes	Neutrophils + basophils + eosinophils + CD66b- neutrophils	36.3	31.9	3.41	3.26	17.0	8.23	19.8
**Neutrophils**	CD66b dim,+ CD16+ HLA-DR-	34.3	28.6	1.67	1.60	16.4	5.58	18.7
**Basophils**	HLA-DR- CD66b- CD123 dim,+ CD38+ CD294+	0.91	1.46	1.12	1.08	0.04	2.12	0
**Eosinophils**	CD14- CD3- CD19- HLA-DR- CD294+ CD66b dim,+	0.87	1.14	0.01	0.02	0.25	0.04	0.04
**$CD66b^-$ neutrophils**	CD3- CD19- CD66b- CD56- HLA-DR- CD123- CD45-	0.29	0.74	0.60	0.57	0.32	0.49	1.06

Table S3. Hematologic data of No. 1 VKH disease patient.

Acute							
**Test Item**	**Result**	**Unit**	**Reference Value**	**Test Item**	**Result**	**Unit**	**Reference Value**
**Blood count**				**Endocrine**			
RBC	4.14	x 10^6 /μL	4.35 ～ 5.55	sIL-2R	261	U/mL	157 ～ 474
Hb	13.4	g/dL	13.7 ～ 16.8	**Immunity**			
Ht	41.6	%	40.0 ～ 50.0	CRP	≦ 0.3	mg/dL	≦ 0.3
MCV	100.5	fL	83.6 ～ 98.2	Toxoplasma IgG	< 1.6	IU/mL	< 1.6
MCH	32.4	pg	27.5 ～ 33.2	Toxoplasma IgM	0.14	S/CO	< 0.83
MCHC	32.2	%	31.7 ～ 35.3	IgG	1048	mg/dL	870 ～ 1700
RDW	12.8	fL	11.6 ～ 14.0	IgA	199	mg/dL	110 ～ 410
WBC	6.50	x 10^3 /μL	3.3 ～ 8.6	IgM	36	mg/dL	35 ～ 220
Neutrophil	63.2	%	38.5 ～ 80.5	**Infection**			
Lymphocyte	28.0	%	16.5 ～ 49.5	RPR	(−)		(−)
MONO	6.3	%	2.0 ～ 10.0	TPHA	(−)		(−)
EOSINO	1.9	%	0 ～ 8.5	HBsAg	(−)		(−)
BASO	0.6	%	0 ～ 2.5	Anti-HCV Ab	0.1		< 0.1
Platelet	316	x 10^3 /μL	158 ～ 348	Anti-HIV Ab			(−)
**Biochemistry**				Anti-HSV Ab	< 4	times	< 4
T-Bil	0.50	mg/dL	0.2 ～ 1.2	Anti-VZV Ab	8	times	< 4
AST	21	U/L	8 ～ 30	Anti-CMV Ab	< 4	times	< 4
ALT	13	U/L	5 ～ 35	**Coagulation**			
LDH	191	U/L	100 ～ 225	PT			
Total protein	7.0	mg/dL	6.5 ～ 8.2	Time	10.3	sec	9.9 ～ 11.8
FBS	89	mg/dL	65 ～ 110	Activity	108.3	%	80 ～ 127
HbA1c	5.4	%	4.6 ～ 6.2	PT-INR	0.96		0.9 ～ 1.1
BUN	14	mg/dL	8 ～ 20	APTT			
Creatinine	0.77	mg/dL	0.61 ～ 1.13	Time	28.1	sec	24.0 ～ 32.0
Na	141	mmol/L	135 ～ 147	Fibrinogen	339	mg/dL	180 ～ 400
K	4.1	mmol/L	3.5 ～ 5.0	ESR (1 hour)	15	mm	1 ～ 7
Cl	102	mmol/L	98 ～ 108				
Ca	9.4	mmol/L	8.5 ～ 10.3				
ACE	7.4	IU/L	7.7 ～ 29.4				
eGFR	77.6	mL/min/1.73m^2					

Remission							
**Test item**	**Result**	**Unit**	**Reference value**	**Test item**	**Result**	**Unit**	**Reference value**
**Blood count**				**Endocrine**			
RBC	4.14	x 10^6 /μL	4.35 ～ 5.55	sIL-2R	261	U/mL	157 ～ 474
Hb	13.4	g/dL	13.7 ～ 16.8	**Immunity**			
Ht	41.6	%	40.0 ～ 50.0	CRP	≦ 0.3	mg/dL	≦ 0.3
MCV	100.5	fL	83.6 ～ 98.2	Toxoplasma IgG	< 1.6	IU/mL	< 1.6
MCH	32.4	pg	27.5 ～ 33.2	Toxoplasma IgM	0.14	S/CO	< 0.83
MCHC	32.2	%	31.7 ～ 35.3	IgG	1048	mg/dL	870 ～ 1700
RDW	12.8	fL	11.6 ～ 14.0	IgA	199	mg/dL	110 ～ 410
WBC	6.50	x 10^3 /μL	3.3 ～ 8.6	IgM	36	mg/dL	35 ～ 220
Neutrophil	63.2	%	38.5 ～ 80.5	**Infection**			
Lymphocyte	28.0	%	16.5 ～ 49.5	RPR	(−)		(−)
MONO	6.3	%	2.0 ～ 10.0	TPHA	(−)		(−)

EOSINO	1.9	%	0 ～ 8.5	HBsAg	(−)		(−)
BASO	0.6	%	0 ～ 2.5	Anti-HCV Ab	0.1		< 0.1
Platelet	316	x 10^3 /μL	158 ～ 348	Anti-HIV Ab			(−)
**Biochemistry**				Anti-HSV Ab	< 4	times	< 4
T-Bil	0.50	mg/dL	0.2 ～ 1.2	Anti-VZV Ab	8	times	< 4
AST	21	U/L	8 ～ 30	Anti-CMV Ab	< 4	times	< 4
ALT	13	U/L	5 ～ 35	**Coagulation**			
LDH	191	U/L	100 ～ 225	PT			
Total protein	7.0	mg/dL	6.5 ～ 8.2	Time	10.3	sec	9.9 ～ 11.8
FBS	89	mg/dL	65 ～ 110	Activity	108.3	%	80 ～ 127
HbA1c	5.4	%	4.6 ～ 6.2	PT-INR	0.96		0.9 ～ 1.1
BUN	14	mg/dL	8 ～ 20	APTT			
Creatinine	0.77	mg/dL	0.61 ～ 1.13	Time	28.1	sec	24.0 ～ 32.0
Na	141	mmol/L	135 ～ 147	Fibrinogen	339	mg/dL	180 ～ 400
K	4.1	mmol/L	3.5 ～ 5.0	ESR (1 hour)	15	mm	1 ～ 7
Cl	102	mmol/L	98 ～ 108				
Ca	9.4	mmol/L	8.5 ～ 10.3				
ACE	7.4	IU/L	7.7 ～ 29.4				
eGFR	77.6	mL/min/1.73m^2					

IgG and IgM levels of toxoplasma are determined by enzyme immunoassay. ACE; angiotensin converting enzyme, ALT; alanine aminotransferase, APTT; activated partial thromboplastin time, AST; aspartate transaminase, BUN; blood urea nitrogen, Ca; calcium, Cl; chlorine, CMV; cytomegalovirus, CRP; C-reactive protein, eGFR; estimated glomerular filtration rate, ESR; erythrocyte sedimentation rate, FBS; fasting blood sugar, Hb; hemoglobin, HBsAg; hepatitis B surface antigen, HCV; hepatitis C virus, HIV; human immunodeficiency virus, HSV; herpes simplex virus, Ht; hematocrit, Ig; immunoglobulin, K; potassium, LDH; lactate dehydrogenase, Na; sodium, MCH; mean corpuscular hemoglobin, MCHC; mean corpuscular hemoglobin concentration, MCV; mean corpuscular volume, PT; prothrombin time, PT-INR; prothrombin time-international normalized ratio, RPR; rapid plasma reagin, RBC; red blood cell, RDW; red blood cell distribution width, sIL-2R; soluble interleukin-2 receptor, TPHA; treponema pallidum hemagglutination test, VZV; varicella-zoster virus, WBC; white blood cells.

Table S4. Hematologic data of No. 2 VKH disease patient.

Acute							
**Test item**	**Result**	**Unit**	**Reference value**	**Test item**	**Result**	**Unit**	**Reference value**
**Blood count**				**Endocrine**			
RBC	4.29	x 10^6/μL	4.35 ～ 5.55	sIL-2R	383	U/mL	157 ～ 474
Hb	14.2	g/dL	13.7 ～ 16.8	**Immunity**			
Ht	43.2	%	40.0 ～ 50.0	CRP	≦ 0.3	mg/dL	≦ 0.3
MCV	100.7	fL	83.6 ～ 98.2	Toxoplasma IgG	(−)	IU/mL	< 1.6
MCH	33.1	pg	27.5 ～ 33.2	Toxoplasma IgM	(−)	S/CO	< 0.83
MCHC	32.9	%	31.7 ～ 35.3	IgG	1129	mg/dL	870 ～ 1700
RDW	13.2	fL	11.6 ～ 14.0	IgA	158	mg/dL	110 ～ 410
WBC	6.10	x 10^3 /μL	3.3 ～ 8.6	IgM	80	mg/dL	35 ～ 220
Neutrophil	55.6	%	38.5 ～ 80.5	**Infection**			
Lymphocyte	35.6	%	16.5 ～ 49.5	RPR	(−)		(−)
MONO	5.7	%	2.0 ～ 10.0	TPHA	(−)		(−)
EOSINO	2.6	%	0 ～ 8.5	HBsAg	(−)		(−)
BASO	0.5	%	0 ～ 2.5	Anti-HCV Ab	0.1		< 0.1
Platelet	258	x 10^3 /μL	158 ～ 348	Anti-HIV Ab			(−)
**Biochemistry**				Anti-HSV Ab	32	times	< 4
T-Bil	0.59	mg/dL	0.2 ～ 1.2	Anti-VZV Ab	8	times	< 4
AST	21	U/L	8 ～ 30	Anti-CMV Ab	< 4	times	< 4
ALT	18	U/L	5 ～ 35	**Coagulation**			
LDH	208	U/L	100 ～ 225	PT			
Total protein	7.3	mg/dL	6.5 ～ 8.2	Time	10.8	sec	9.9 ～ 11.8
FBS	96	mg/dL	65 ～ 110	Activity	97.5	%	80 ～ 127
HbA1c	5.8	%	4.6 ～ 6.2	PT-INR	1.01		0.9 ～ 1.1
BUN	9	mg/dL	8 ～ 20	APTT			
Creatinine	0.66	mg/dL	0.61 ～ 1.13	Time	30.9	sec	24.0 ～ 32.0
Na	143	mmol/L	135 ～ 147	Fibrinogen	404	mg/dL	180 ～ 400
K	3.9	mmol/L	3.5 ～ 5.0	ESR (1 hour)	32	mm	1 ～ 7
Cl	106	mmol/L	98 ～ 108				
Ca	9.4	mmol/L	8.5 ～ 10.3				
CK	77	U/L	≦160				
ACE	12.6	IU/L	7.7 ～ 29.4				
eGFR	71.9	mL/min/1.73m^2					

Remission							
**Test item**	**Result**	**Unit**	**Reference value**	**Test item**	**Result**	**Unit**	**Reference value**
**Blood count**				**Immunity**			
RBC	4.15	x 10^6/μL	4.35 ～ 5.55	CRP	0.7	mg/dL	≦ 0.3
Hb	13.9	g/dL	13.7 ～ 16.8				
Ht	42.5	%	40.0 ～ 50.0				
MCV	102.4	fL	83.6 ～ 98.2				
MCH	33.5	pg	27.5 ～ 33.2				
MCHC	32.7	%	31.7 ～ 35.3				
RDW	12.6	fL	11.6 ～ 14.0				
WBC	5.00	x 10^3 /μL	3.3 ～ 8.6				
Neutrophil	61.6	%	38.5 ～ 80.5				
Lymphocyte	25	%	16.5 ～ 49.5				

MONO	11.6	%	2.0 ～ 10.0
EOSINO	1.6	%	0 ～ 8.5
BASO	0.2	%	0 ～ 2.5
Platelet	234	x 10^3 /μL	158 ～ 348
**Biochemistry**			
T-Bil	0.30	mg/dL	0.2 ～ 1.2
AST	24	U/L	8 ～ 30
LDH	228	U/L	100 ～ 225
ALP	69	U/L	38 ～ 113
Total protein	6.3	mg/dL	6.5 ～ 8.2
BUN	23	mg/dL	8 ～ 20
Creatinine	0.82	mg/dL	0.61 ～ 1.13
Na	142	mmol/L	135 ～ 147
K	3.6	mmol/L	3.5 ～ 5.0
Cl	106	mmol/L	98 ～ 108
Ca	9.4	mmol/L	8.5 ～ 10.3

CK; creatine kinase.

Table S5. Hematologic data of No. 3 VKH disease patient.

Acute							
**Test item**	**Result**	**Unit**	**Reference value**	**Test item**	**Result**	**Unit**	**Reference value**
**Blood count**				**Immunity**			
RBC	4.42	x 10^6 /μL	4.35 ～ 5.55	CRP	≦ 0.3	mg/dL	≦ 0.3
Hb	13.1	g/dL	13.7 ～ 16.8	Toxoplasma IgG	< 1.6	IU/mL	< 1.6
Ht	40.3	%	40.0 ～ 50.0	Toxoplasma IgM	0.14	S/CO	< 0.83
MCV	91.2	fL	83.6 ～ 98.2	IgG	1256	mg/dL	870 ～ 1700
MCH	29.6	pg	27.5 ～ 33.2	IgA	519	mg/dL	110 ～ 410
MCHC	32.5	%	31.7 ～ 35.3	IgM	52	mg/dL	35 ～ 220
RDW	12.2	fL	11.6 ～ 14.0	**Infection**			
WBC	5.50	x 10^3 /μL	3.3 ～ 8.6	RPR	(−)		(−)
Neutrophil	60.6	%	38.5 ～ 80.5	TPHA	(−)		(−)
Lymphocyte	32.4	%	16.5 ～ 49.5	HBsAg	(−)		(−)
MONO	5.2	%	2.0 ～ 10.0	Anti-HCV Ab	0.1		< 0.1
EOSINO	1.4	%	0 ～ 8.5	Anti-HIV Ab			(−)
BASO	0.4	%	0 ～ 2.5	Anti-HSV Ab	＜4	times	< 4
Platelet	272	x 10^3 /μL	158 ～ 348	Anti-VZV Ab	8	times	< 4
**Biochemistry**				Anti-CMV Ab	8	times	< 4
T-Bil	1.41	mg/dL	0.2 ～ 1.2	**Coagulation**			
AST	26	U/L	8 ～ 30	PT			
ALT	21	U/L	5 ～ 35	Time	10.1	sec	9.9 ～ 11.8
LDH	221	U/L	100 ～ 225	Activity	103.0	%	80 ～ 127
Total protein	7.8	mg/dL	6.5 ～ 8.2	PT-INR	0.99		0.9 ～ 1.1
FBS	93	mg/dL	65 ～ 110	APTT			
HbA1c	5.9	%	4.6 ～ 6.2	Time	28.1	sec	24.0 ～ 32.0
BUN	18	mg/dL	8 ～ 20	Fibrinogen	310	mg/dL	180 ～ 400
Creatinine	0.95	mg/dL	0.61 ～ 1.13	ESR (1 hour)	29	mm	1 ～ 7
Na	143	mmol/L	135 ～ 147				

K	4.3	mmol/L	3.5 ～ 5.0
Cl	105	mmol/L	98 ～ 108
Ca	10.1	mmol/L	8.5 ～ 10.3
ACE	21	IU/L	7.7 ～ 29.4
eGFR	43.9	mL/min/1.73m^2	

Remission							
**Test item**	**Result**	**Unit**	**Reference value**	**Test item**	**Result**	**Unit**	**Reference value**
**Blood count**				**Endocrine**			
RBC	4.33	x 10^6 /μL	4.35 ～ 5.55	sIL-2R	261	U/mL	157 ～ 474
Hb	13.3	g/dL	13.7 ～ 16.8	**Immunity**			
Ht	40	%	40.0 ～ 50.0	CRP	≦0.14	mg/dL	≦ 0.3
MCV	92.4	fL	83.6 ～ 98.2	**Infection**			
MCH	30.7	pg	27.5 ～ 33.2	RPR	(−)		(−)
MCHC	33.3	%	31.7 ～ 35.3	TPHA	(−)		(−)
RDW	11.9	fL	11.6 ～ 14.0	HBsAg	(−)		(−)
WBC	7.00	x 10^3 /μL	3.3 ～ 8.6	Anti-HCV Ab	0.1		< 0.1
Neutrophil	77.5	%	38.5 ～ 80.5	Anti-HIV Ab	(−)		(−)
Lymphocyte	20.2	%	16.5 ～ 49.5	**Coagulation**			
MONO	1.9	%	2.0 ～ 10.0	PT			
EOSINO	0.3	%	0 ～ 8.5	Time	10.3	sec	9.9 ～ 11.8
BASO	0.1	%	0 ～ 2.5	Activity	114.2	%	80 ～ 127
Platelet	239	x 10^3 /μL	158 ～ 348	PT-INR	0.94		0.9 ～ 1.1
**Biochemistry**				APTT			
T-Bil	1.30	mg/dL	0.2 ～ 1.2	Time	25.8	sec	24.0 ～ 32.0
AST	27	U/L	8 ～ 30				
ALT	21	U/L	5 ～ 35				
LDH	276	U/L	100 ～ 225				
FBS	103	mg/dL	65 ～ 110				
HbA1c	6.0	%	4.6 ～ 6.2				
BUN	19	mg/dL	8 ～ 20				
Creatinine	0.93	mg/dL	0.61 ～ 1.13				
Na	141	mmol/L	135 ～ 147				
K	4.2	mmol/L	3.5 ～ 5.0				
Cl	106	mmol/L	98 ～ 108				
eGFR	44.8	mL/min/1.73m^2					

Table S6. Serum cytokine levels in each VKH disease patient.

**Category**	**VKH**						**Detection range**
**Disease stage**	**Acute**			**Remission**			
**Patient number**	**No. 1**	**No. 2**	**No. 3**	**No. 1**	**No. 2**	**No. 3**	
PDGF-BB	1255.7	118.9	345.4	1232.0	54.0	288.4	7.67 to 42619.2
IL-1β	0.54	0.39	0	0	0	0	0.28 to 5036.8
IL-1ra	24.0	21.6	36.1	31.2	42.1	36.1	8.56 to 37276.7
IL-2	0	0	0	0	0	0	1.50 to 8778.3
IL-4	1.63	1.22	4.16	3.46	3.44	4.69	0.16 to 3539.9
IL-5	0	0	0	0	0	0	4.02 to 85349.0
IL-6	0	0	0	0	0	0	0.37 to 5969.6
IL-7	33.7	51.7	0	0	25.6	0	1.84 to 36229.8
IL-8	0	0	2.55	1.30	1.50	2.82	0.51 to 10416.3
IL-9	27.4	20.9	49.1	44.4	47.2	43.2	0.92 to 21827.0
IL-10	0	39.8	0	0	18.4	0	0.84 to 12755.7
IL-12	0	24.9	0	0	0	0	1.58 to 21263.1

IL-13	0	4.36	0	0	0	0	0.75	to	5003.2
IL-15	0	0	0	0	0	0	####	to	76422.6
IL-17A	5.20	3.38	0	0	3.05	4.21	2.65	to	35294.2
Eotaxin	139.6	98.4	196.6	168.8	19.4	191.5	0.09	to	1487.0
bFGF	5.99	4.94	0	0	0	0	3.52	to	5445.8
G-CSF	0	519.8	142.8	99.3	362.0	166.5	55.0	to	70102.6
GM-CSF	0	0	0	0	0	0	0.33	to	1822.9
IFN-γ	0	0	0	0	0	1.75	0.74	to	22825.9
IP-10	542.8	847.2	153.0	52.3	70.5	133.0	1.53	to	23765.1
MCP-1	5.10	4.25	8.45	8.78	7.16	5.80	0.34	to	5761.9
MIP-1α	0.19	0	1.18	0.53	0.45	2.37	0.05	to	348.6
MIP-1β	27.1	22.6	26.3	18.8	23.0	36.6	0.46	to	2126.1
RANTES	1010.2	937.3	748.6	649.4	210.7	580.3	1.00	to	5544.2
TNFα	15.5	0	7.32	21.1	0	13.4	2.73	to	53796.5
VEGF-A	0	0	0	0	0	0	19.5	to	69174.0

bFGF; basic fibroblast growth factor, G-CSF; granulocyte colony-stimulating factor, GM-CSF; granulocyte macrophage colony-stimulating factor, IFN; interferon, IP-10; interferon gamma-induced protein 10, IL; interleukin, MIP; macrophage inflammatory protein, MCP; monocyte chemotactic protein, PDGF; platelet derived growth factor, ra; receptor antagonist, RANTES; regulated on activation, normal T-cell expressed and secreted, TNF; tumor necrosis factor, VEGF; vascular endothelial growth factor.

Table S7. Serum cytokine levels in each control.

Category	Controls							Detection Range		
Reference Number	No. 1	No. 2	No. 3	No. 4	No. 5	No. 6	No. 7			
PDGF-BB	83.6	36.8	171.9	360.4	1491.9	1113.4	506.8	7.67	to	42619.2
IL-1β	0.31	0	0	1.08	0.42	0.74	0	0.28	to	5036.8
IL-1ra	0	0	0	41.9	13.1	45.3	22.3	8.56	to	37276.7
IL-2	0	0	0	0	0	0	0	1.50	to	8778.3
IL-4	0.27	0	0	2.07	1.36	2.13	2.03	0.16	to	3539.9
IL-5	0	0	0	0	0	0	0	4.02	to	85349.0
IL-6	0	0	0	0	0	0	0	0.37	to	5969.6
IL-7	0	0	0	33.2	18.1	16.9	0	1.84	to	36229.8
IL-8	0	0	0	0	0	17.6	1.10	0.51	to	10416.3
IL-9	0	0	0	45.6	2.89	35.9	45.6	0.92	to	21827.0
IL-10	0	0	0	0	0	0	0	0.84	to	12755.7
IL-12	0	0	0	0	0	0	0	1.58	to	21263.1
IL-13	0	0	0	0	0	0	0	0.75	to	5003.2
IL-15	0	0	0	0	0	0	0	####	to	76422.6
IL-17A	0	0	0	5.92	0	6.97	0	2.65	to	35294.2
Eotaxin	7.60	4.04	4.54	110.5	57.5	139.7	9.29	0.09	to	1487.0
bFGF	4.67	0	0	7.22	4.67	5.21	0	3.52	to	5445.8
G-CSF	0	0	0	0	0	0	111.1	55.0	to	70102.6
GM-CSF	0	0	0	0	0	0	0	0.33	to	1822.9
IFN-γ	0	0	0	0	0	0	0	0.74	to	22825.9
IP-10	48.3	117.7	63.9	778.0	28.5	332.3	53.0	1.53	to	23765.1
MCP-1	0	0	0	3.86	0	0	0.60	0.34	to	5761.9
MIP-1α	0	0	0	0.51	0	0.93	1.62	0.05	to	348.6
MIP-1β	3.41	0	0	38.7	5.78	34.5	31.1	0.46	to	2126.1
RANTES	28.6	46.9	66.9	179.7	488.1	1446.2	93.5	1.00	to	5544.2
TNFα	0	0	0	15.5	0	31.6	0	2.73	to	53796.5
VEGF-A	0	0	0	0	0	0	0	19.5	to	69174.0

Figure S1. Clinical course of No. 1 VKH disease patient under glucocorticoid therapy.

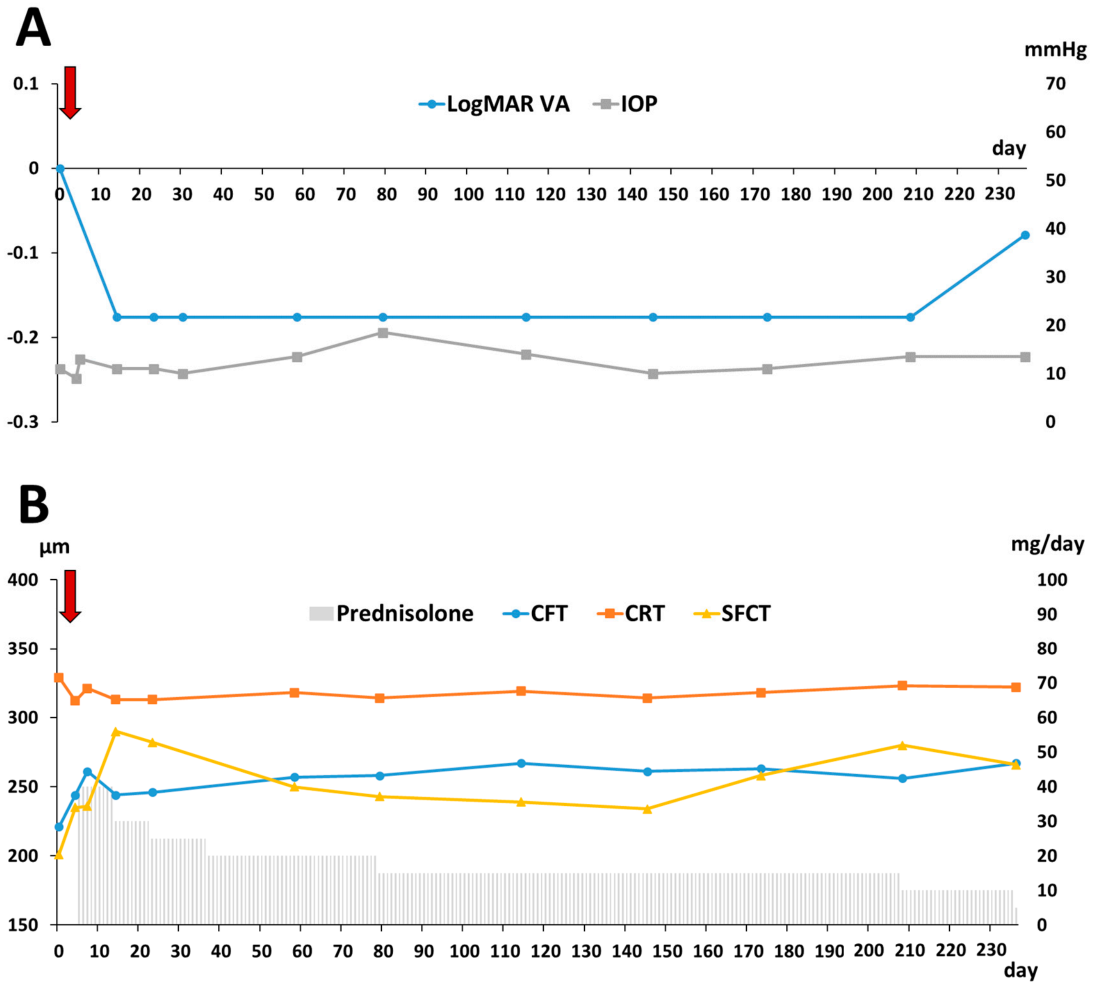

Time courses of (A) logMAR VA, IOP, and (B) dose of prednisolone, CFT, CRT, SFCT in No.1 VKH disease patient under GC therapy are shown. Left vertical axis shows logMAR VA in (A), CFT, CRT, SFCT in (B), and right vertical axis means IOP in (A), dose of prednisolone in (B). Horizontal axis denotes number of days passed since first medical examination. Red arrows in (A, B) indicate intravenous pulses of methylprednisolone (1,000 mg/day for 3 days). CFT; central foveal thickness, CRT; central retinal thickness, GC; glucocorticoid, SFCT; subfoveal choroidal thickness. VKH; Vogt-Koyanagi-Harada.

Figure S2. Clinical course of No. 2 VKH disease patient under glucocorticoid therapy.

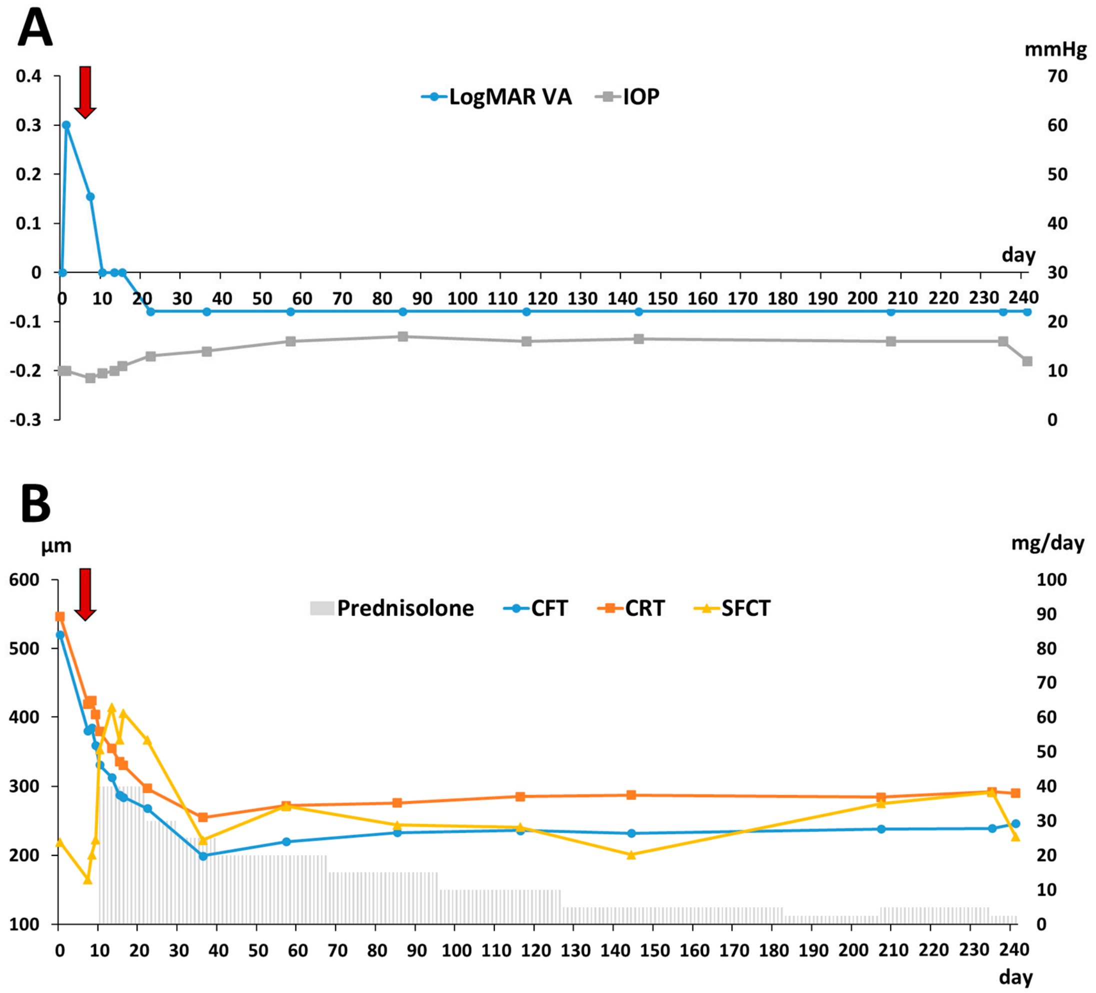

Time courses of (A) logMAR VA, IOP, and (B) dose of prednisolone, CFT, CRT, SFCT in No.2 VKH disease patient under GC therapy are presented.

Figure S3. Clinical course of No. 3 VKH disease patient under glucocorticoid therapy.

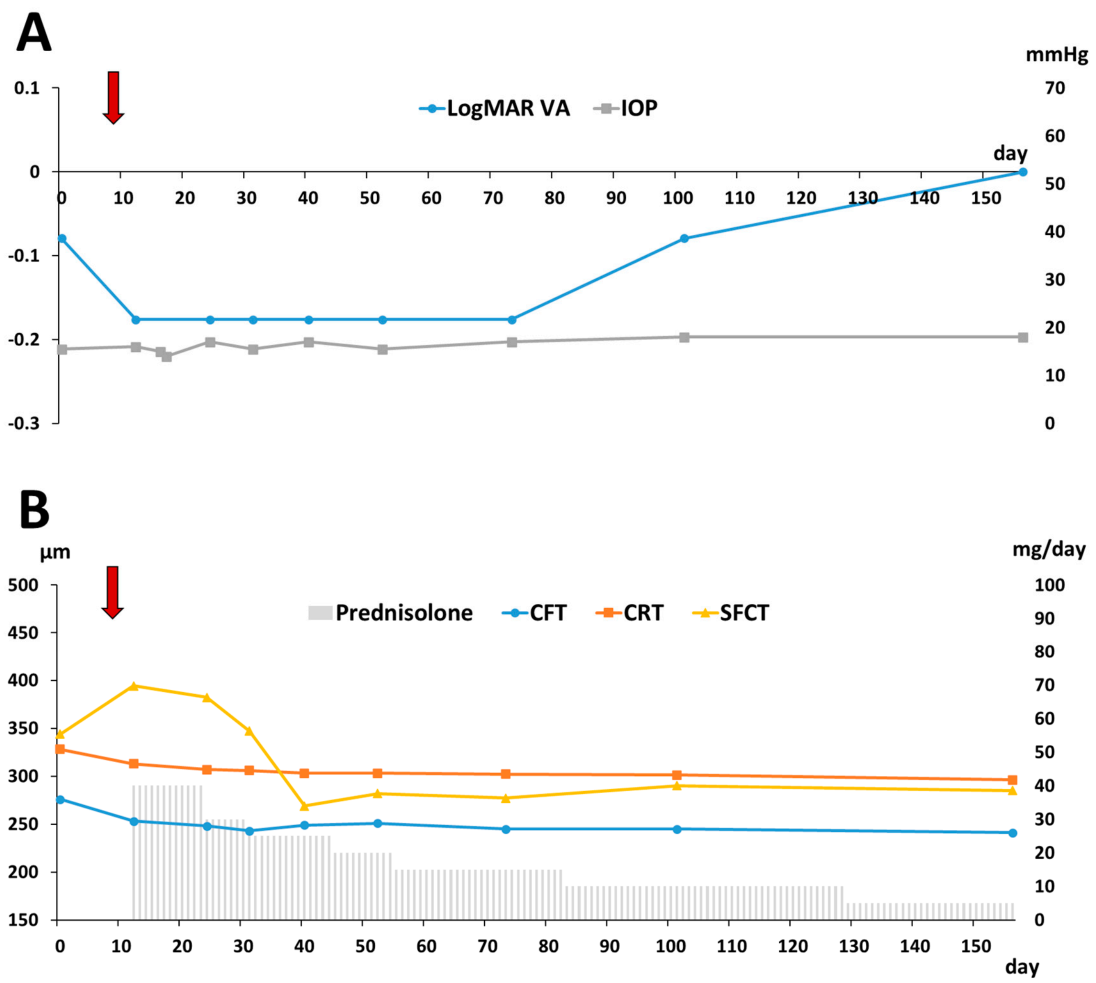

Time courses of (A) logMAR VA, IOP, and (B) dose of prednisolone, CFT, CRT, SFCT in No.3 VKH disease patient under GC therapy are shown.
